# Evaluation of the Impact of Antimicrobial Use Protocols in Porcine Reproductive and Respiratory Syndrome Virus-Infected Swine on Phenotypic Antimicrobial Resistance Patterns

**DOI:** 10.1128/AEM.00970-21

**Published:** 2022-01-11

**Authors:** Carissa A. Odland, Roy Edler, Noelle R. Noyes, Scott A. Dee, Joel Nerem, Peter R. Davies

**Affiliations:** a Pipestone Veterinary Services, Pipestone, Minnesota, USA; b Pipestone Applied Research, Pipestone, Minnesota, USA; c University of Minnesota, Saint Paul, Minnesota, USA; Centers for Disease Control and Prevention

**Keywords:** pig, antimicrobial resistance, *E. coli*, *Enterococcus*, porcine reproductive and respiratory syndrome virus (PRRSV)

## Abstract

A longitudinal study was conducted to assess the impact of different antimicrobial exposures of nursery-phase pigs on patterns of phenotypic antimicrobial resistance (AMR) in fecal indicator organisms throughout the growing phase. Based on practical approaches used to treat moderate to severe porcine reproductive and respiratory syndrome virus (PRRSV)-associated secondary bacterial infections, two antimicrobial protocols of differing intensities of exposure [44.1 and 181.5 animal-treatment days per 1000 animal days at risk (ATD)] were compared with a control group with minimal antimicrobial exposure (2.1 ATD). Litter-matched pigs (*n* = 108) with no prior antimicrobial exposure were assigned randomly to the treatment groups. Pen fecal samples were collected nine times during the wean-to-finish period and cultured for Escherichia coli and *Enterococcus* spp. Antimicrobial-susceptibility testing was conducted using NARMS Gram-negative and Gram-positive antibiotic panels. Despite up to 65-fold difference in ATD, few and modest differences were observed between groups and over time. Resistance patterns at marketing overall remained similar to those observed at weaning, prior to any antimicrobial exposures. Those differences observed could not readily be reconciled with the patterns of antimicrobial exposure. Resistance of E. coli to streptomycin was higher in the group exposed to 44.1 ATD, but no aminoglycosides were used. In all instances where resistances differed between time points, the higher resistance occurred early in the trial prior to any antimicrobial exposures. These minimal impacts on AMR despite substantially different antimicrobial exposures point to the lack of understanding of the drivers of AMR at the population level and the likely importance of factors other than antimicrobial exposure.

**IMPORTANCE** Despite a recognized need for more longitudinal studies to assess the effects of antimicrobial use on resistance in food animals, they remain sparse in the literature, and most longitudinal studies of pigs have been observational. The current experimental study had the advantages of greater control of potential confounding, precise measurement of antimicrobial exposures which differed markedly between groups and tracking of pigs until market age. Overall, resistance patterns were remarkably stable between the treatment groups over time, and the differences observed could not be readily reconciled with the antimicrobial exposures, indicating the likely importance of other determinants of AMR at the population level.

## INTRODUCTION

In veterinary medicine, antimicrobials are used to combat bacterial pathogens that undermine animal health and well-being. Antimicrobial resistance (AMR) may impact both animal health, through loss of efficacy of antimicrobials used to treat animal pathogens, and human health via transmission of resistant organisms from animals to people (1). The contribution of antimicrobial use in animals to the burden of AMR in human medicine remains a subject of longstanding and unresolved debate (2–5). Although it is now established that companion animals can also serve as sources of human infections with resistant bacteria (6–8), most attention has been focused on food animal populations.

Regardless of the ongoing uncertainties surrounding the potential impact upon public health, veterinarians should seek to refine and optimize their prescribing practices. An evidence-based approach to antimicrobial stewardship requires understanding of the impact that current practices of antimicrobial use may have on both animal health and AMR. Understanding these impacts is not a trivial undertaking, as antimicrobial use encompasses treating diverse clinical diseases with various antimicrobial compounds that may be administered by several routes and a myriad of doses and durations. Potential resistance outcomes include both phenotypic and genotypic dimensions that may be assessed in a small number of selected bacterial species, or more broadly across the microbiome. In food animals, one aspect that has been largely ignored is the extent to which the timing of antimicrobial exposure of food animals in relation to harvest may influence the profile of resistant pathogens entering the food supply chain. In commercial swine production, the need for antimicrobials is typically greatest in weaned pig populations (9–12), which means that much exposure to antimicrobials occurs several months prior to marketing. The need for more longitudinal studies, rather than cross-sectional studies, to assess the effects of antimicrobial use in food animals is recognized (13, 14).

Experimental and field studies over relatively short intervals (hours to several weeks) have reported increased detection of antimicrobial resistant bacteria or genes following antimicrobial exposure of pigs or pig feces (13, 15–21). Longitudinal studies over intervals approximating the usual growth period (around 6 months in pigs in the USA) are needed to understand the temporal implications of antimicrobial exposures of food animals (13, 14). Previous longitudinal studies of AMR in swine have mostly been observational studies (22), have not reported precise measures of antimicrobial exposure (23, 24), or have not followed defined cohorts of animals until market age (25, 26). A recent study of a cohort of pigs raised on a farrow to finish farm reported that ‘AMR gene prevalence and abundance were not influenced by antibiotic use, either during the production cycle or following whole-herd medication’ (27). In contrast, several resistance outcomes differed between treated and untreated pigs in an observational study of farrow-to-finish cohorts of pigs in Germany (22).

In the USA, the most problematic swine pathogen over the last 30 years has been porcine reproductive and respiratory syndrome virus (PRRSV) (28, 29). The causal arterivirus is highly host-specific for swine and is a key component of the multifactorial Porcine Respiratory Disease Complex (30). Treatment of porcine respiratory disease, commonly involving PRRSV and secondary bacterial infections, is one of the predominant needs for administration of antimicrobials to pigs in the USA (31). Pneumonia caused by PRRSV alone is typically mild, but secondary bacterial infections can lead to severe clinical disease and mortality (32). A previous experimental study from our group reported clinical and performance outcomes in PRRSV challenged pigs treated with different antimicrobial protocols, including an untreated control group (33). Health and performance parameters were improved in the treated groups, even though the control group, for welfare reasons, also required treatment with antimicrobials due to the severity of disease that developed in untreated animals. The clinical expression of PRRSV-associated disease in pigs is extremely variable, in part due to the genetic diversity of the virus (34). Consequently, the need for antimicrobial treatment can be variable, in line with the disease severity.

Given the importance of PRRSV for driving both swine health and antimicrobial use, there is a need to better understand how AMR may be affected by antimicrobial use (AMU) protocols used in PRRS outbreaks. This longitudinal study was undertaken to compare animal health and AMR outcomes of two antimicrobial treatment protocols in PRRSV challenged pigs. The study compared phenotypic resistance prevalence and patterns in Gram-negative (E. coli) and Gram-positive (*Enterococcus* spp.) commensals. Our primary objective was to evaluate how the AMR prevalence in fecal E. coli and *Enterococcus* spp. would be affected by exposure to antimicrobials, and to describe temporal patterns of AMR prevalence from weaning until market.

## RESULTS

### Clinical observations and growth performance.

Of the 108 pigs placed, 106 (98.1%) completed the trial. Two pigs in the Intensive group were euthanized at 8 days and 13 days post-PRRSV challenge after being assessed with severe clinical signs (score C with the Individual Pig Care system). In total, 1,620 IPC assessments were made of pigs in each of the Minimal and Moderate groups, and 1,580 assessments of pigs in the Intensive group (less due to the mortalities). In the Minimal group (no PRRSV challenge), 98.8% of assessments were scored as normal, compared to 90.0% and 91.6% normal scores for the Moderate and Intensive groups (both PRRSV challenged), respectively. Pigs with mild signs of clinical disease (IPC score A) were identified in 12 (0.7%), 131 (8.1%), and 114 (7.2%) assessments of the Minimal, Moderate, and Intensive groups, respectively. Signs of moderate clinical disease (IPC score B) were recorded in 8 (0.5%), 31 (1.9%) and 18 (1.1%) assessments in the Minimal, Moderate, and Intensive groups, respectively. There were no significant differences in average daily weight gain (ADG) among the treatment groups (*P* = 0.12). However, ADG was numerically greater (*P* = 0.05) in the unchallenged pigs (0.93 g per day, SD 0.08) than in the pigs challenged with the PRRSV challenge isolate (0.89 g per day, SD 0.09). These observations were consistent with the occurrence of mild PRRS disease in the exposed pigs.

### Bacteriology and prevalence of antimicrobial resistance.

Of the 324 composite fecal samples cultured, E. coli was isolated from 307 (94.7%) and *Enterococcus* spp. from 292 (90.1%) samples. There were five *Enterococcus* species identified, including *E. hirae* (121 total isolates out of 292: 21% in Minimal, 34% in Moderate, 45% in Intensive group), E. faecium (102 total isolates: 48% Minimal, 29% Moderate, 23% Intensive), E. faecalis (62: 37% Minimal, 32% Moderate, 31% Intensive), *E*. *durans* (6 isolates, three each in the Minimal and Intensive groups) and *E*. *villorum* (one isolate, Intensive group). At the first sampling after weaning (i.e., before any exposure to antimicrobials), 30 of the 33 E. coli isolates were resistant to at least one antimicrobial, with individual isolates resistant to up to 11 antimicrobials. None of the 36 *Enterococcus* isolates at that sampling were pansusceptible, with individual isolates resistant to between two and nine antimicrobials. Summary MIC results from all sampling periods for E. coli and *Enterococcus* are presented in Tables S1 and S2, respectively.

**FIG 1:**
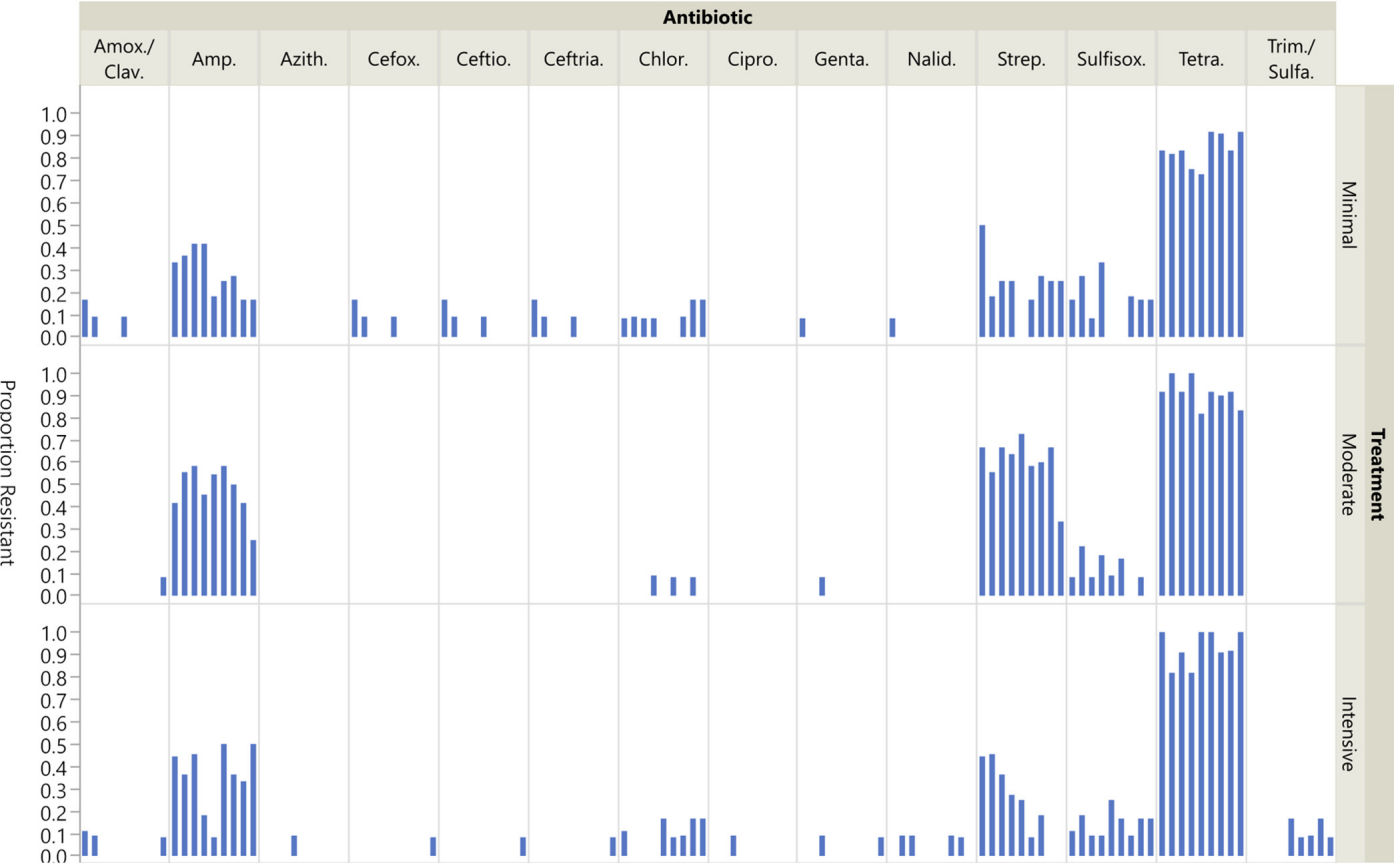
Proportion of E. coli: isolates exhibiting resistance by treatment group and antimicrobial across all sampling time points. Blue bars indicate the prevalence of resistance to the respective antimicrobial in each treatment group (N = 12). Each bar represents a sampling in chronological order at 1-, 6-, 7-, 8-, 9-, 10-, 11-, 14-, and 25-weeks postweaning. Absence of a blue bar indicates susceptibility of all isolates. Prevalence and confidence intervals are presented in Table S3 in the supplemental material. *Antimicrobial key:* Amox/Clav = amoxicillin-clavulanic acid, Amp = ampicillin, Azith = azithromycin, Cefox = cefoxitin, Ceftio = ceftiofur, Ceftria = ceftriaxone, Chlor = chloramphenicol, Cipro = ciprofloxacin, Genta = gentamicin, Nalid = nalidixic acid, Strep = streptomycin, Sulfisox = sulfisoxazole, Tetra = tetracycline, Trim/Sulfa = trimethoprim-sulfamethoxazole.

Among all fecal E. coli isolates, resistance was most common to tetracycline, ampicillin, and streptomycin (Fig. 1). No significant differences in prevalence of resistance in E. coli isolates were observed between different sampling dates. The sole significant finding for E. coli isolates was with streptomycin between treatment groups. The odds of resistance to streptomycin were significantly higher (*P* < 0.0001) in the Moderate group than in the Minimal (OR = 5.45 (CI 2.79–10.68) and Intensive (OR = 5.70 (CI 2.6–12.49,) groups, which did not differ significantly (OR = 0.96 (CI 0.38–2.40, *P* = 0.99).

**FIG 2:**
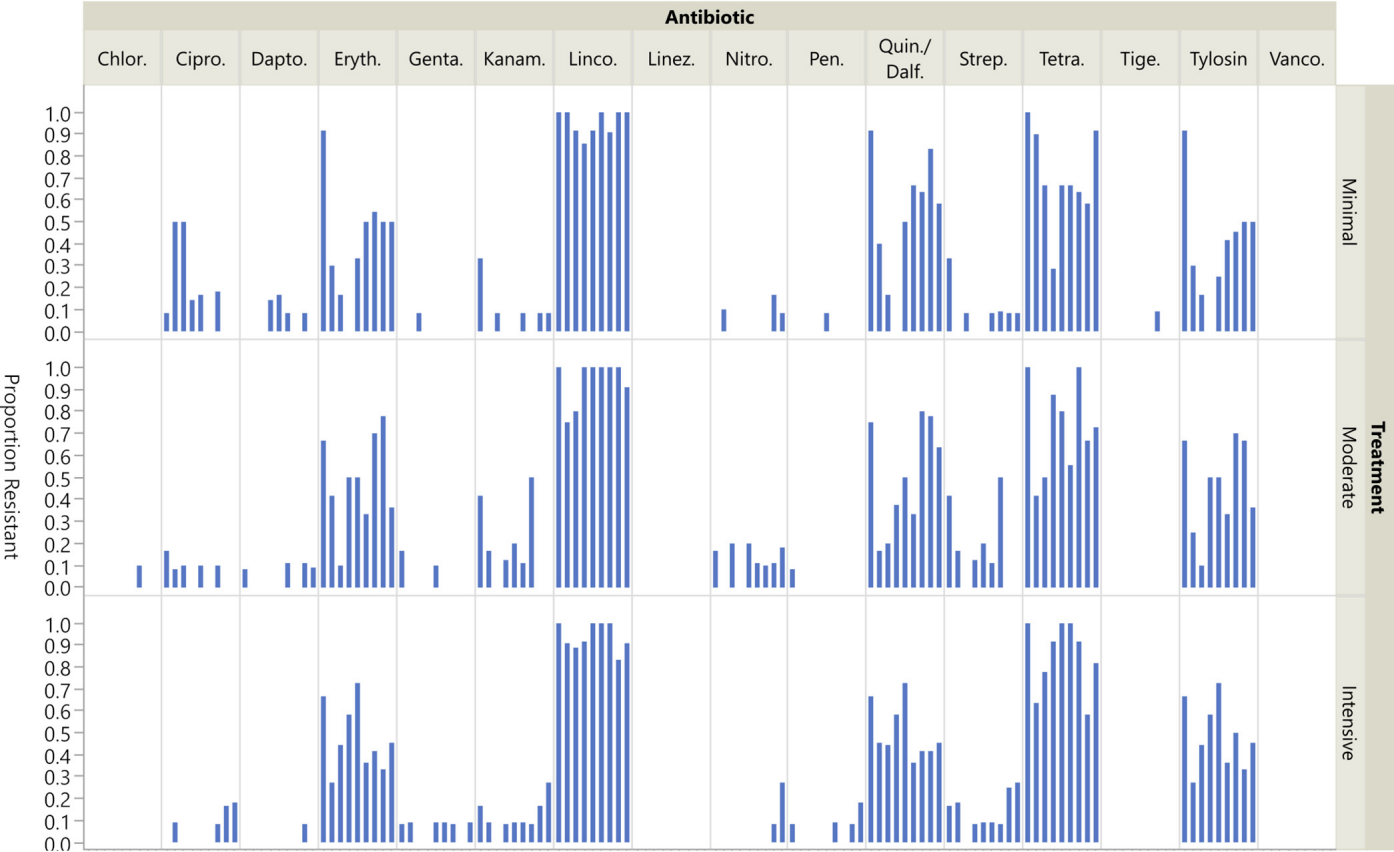
Proportion of *Enterococcus* isolates exhibiting resistance by treatment group and antimicrobial across all sampling time points. Blue bars indicate the prevalence of resistance to the respective antimicrobial in each treatment group (N = 12). Each bar represents a sampling in chronological order at 1-, 6-, 7-, 8-, 9-, 10-, 11-, 14-, and 25-weeks postweaning. Absence of a blue bar indicates susceptibility of all isolates. Prevalence and confidence intervals are presented in Table S3 in the supplemental material. *Antimicrobial key:* Chlor = chloramphenicol, Cipro = ciprofloxacin, Dapto = daptomycin, Eryth = erythromycin, Genta = gentamicin, Kanam = kanamycin, Linco = lincomycin, Linez = linezolid, Nitro = nitrofurantoin, Pen = penicillin, Quin/Dalf = quinupristin-dalfopristin, Strep = streptomycin, Tetra = tetracycline, Tige = tigecycline, Tylosin = tylosin tartrate, Vanco = vancomycin.

Among the *Enterococcus* isolates, resistance was most common to erythromycin, lincomycin, quinupristin-dalfopristin, tetracycline, and tylosin (Fig. 2). No significant differences were identified between treatment groups. Erythromycin, quinupristin-dalfopristin, and tylosin were the only antimicrobials for which significant differences between sampling dates were found for *Enterococcus* spp. The odds of resistance to erythromycin were less in week 6 compared to week 1 (OR = 0.165 [CI 0.058–0.468, *P* = 0.02]), and less in week 7 compared to week 1 (OR = 0.097 [CI 0.028–0.335, *P* = 007]). The likelihood of quinupristin-dalfopristin resistance was less in week 6 compared to week 1 (OR = 0.143 [CI 0.052–0.395, *P* = 0.005]), and less in week 7 compared to week 1 (OR = 0.098 [CI 0.029–0.333, *P* = 0.006]). The odds of resistance to tylosin were less in week 6 compared to week 1 (OR = 0.122 [CI 0.043–0.345, *P* = 0.002]), and less in week 7 compared to week 1 (OR = 0.097 [CI 0.028–0.335, *P* = 0.007]). No significant differences in odds of resistance to erythromycin, quinupristin-dalfopristin, or tylosin were found in comparing other sampling events to Week 1.

### Patterns of antimicrobial resistance.

**FIG 3:**
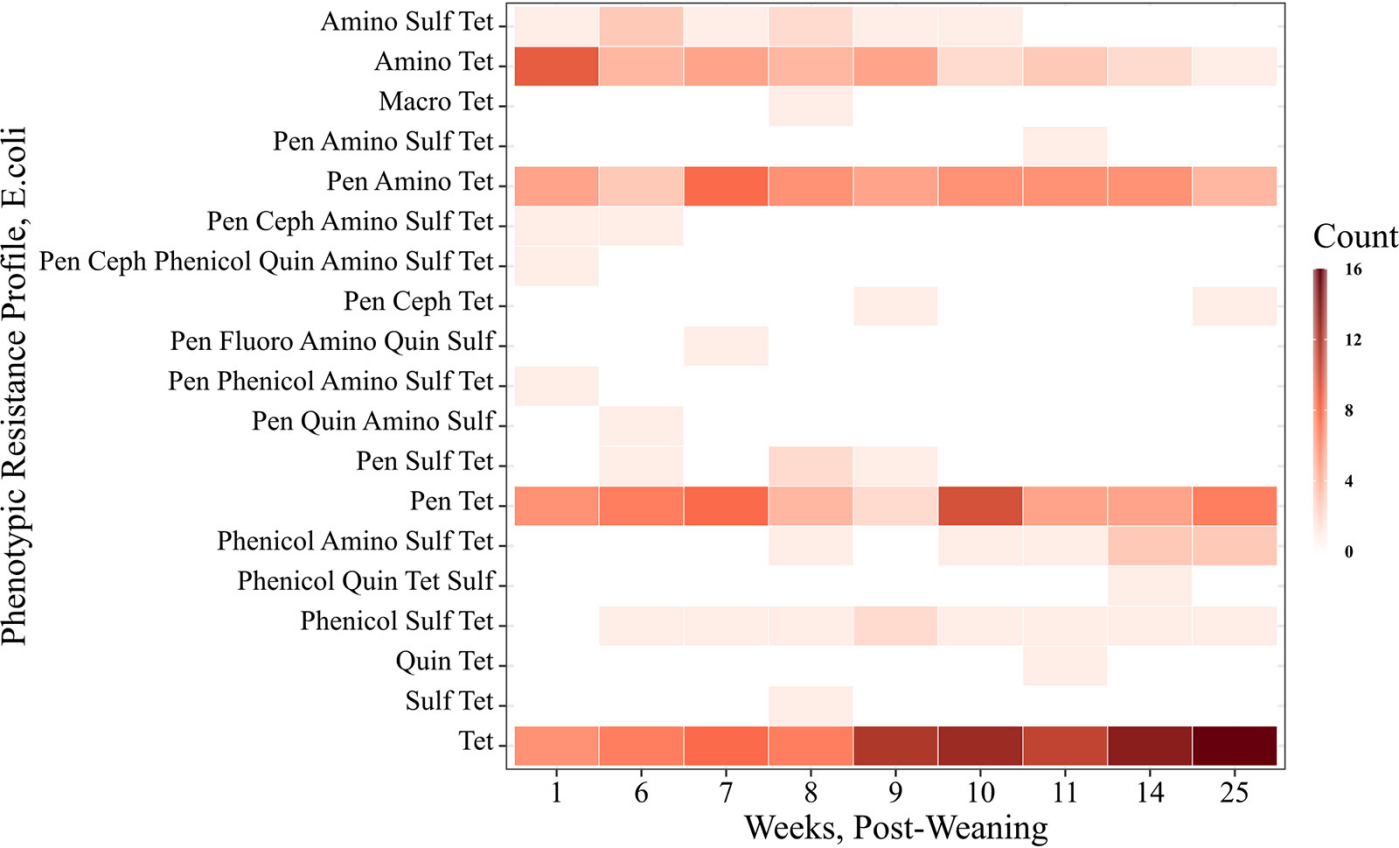
Distribution of antimicrobial resistance profiles of 307 E. coli isolates according to antimicrobial class across each sampling time point. Class key: aminoglycoside = Amino, sulfonamide = Sulf, tetracycline = Tet, macrolide = Macro, phenicol = Phenicol, penicillins = Pen, cephalosporin = Ceph, quinolone = Quin, fluoroquinolone = Fluoro.

Among the 307 E. coli isolates, 30 isolates (10.1%) were pansusceptible to the Gram-negative panel of antimicrobials; and 94 isolates (30.6%) were resistant to tetracycline alone. Of the 182 E. coli isolates resistant to more than one antimicrobial, 19 different resistance profiles were observed. These resistance profiles most frequently included resistance to tetracyclines, penicillins (amoxicillin-clavulanic acid and ampicillin), and aminoglycosides (gentamicin and streptomycin) (Fig. 3).

**FIG 4:**
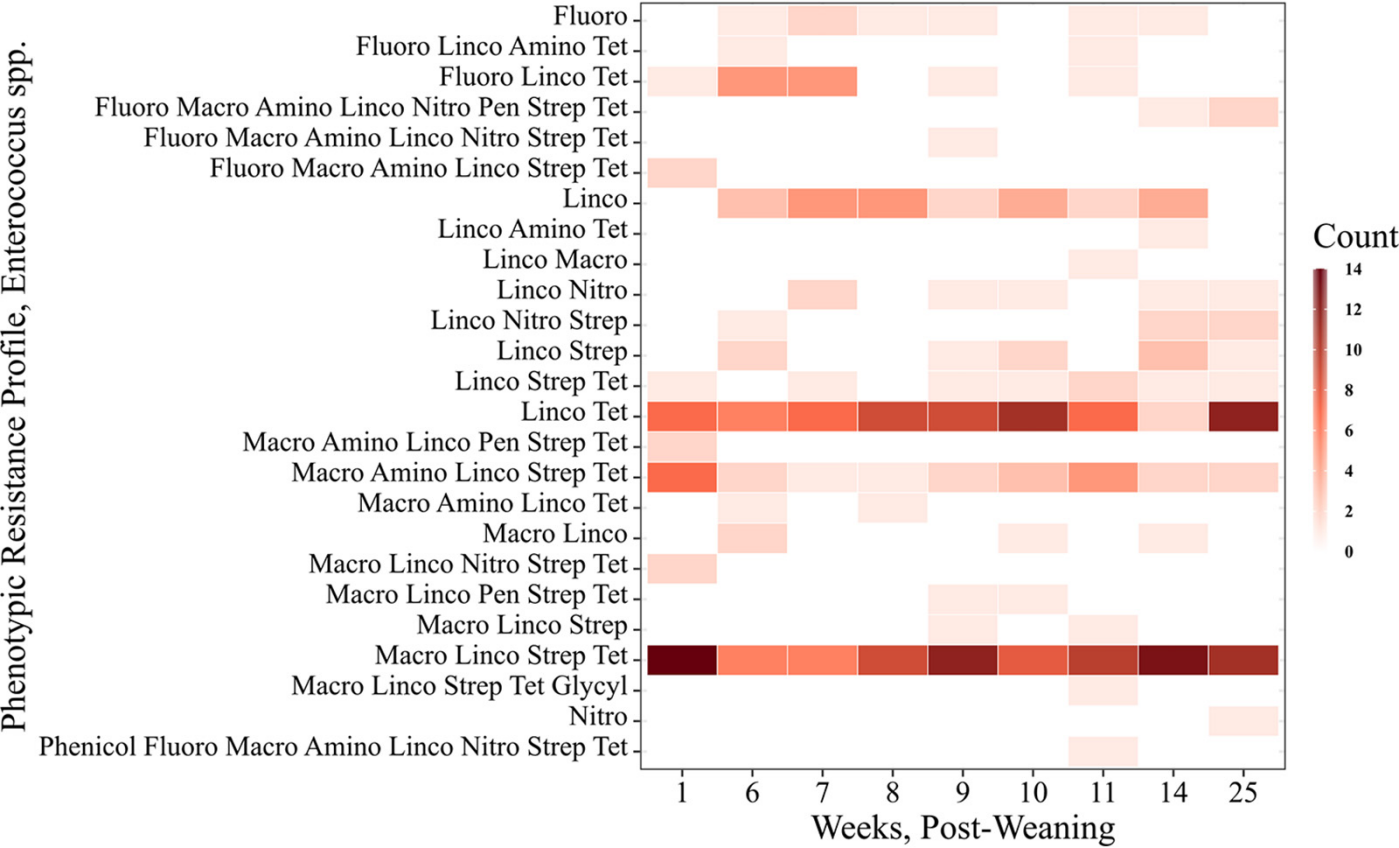
Distribution of antimicrobial resistance profiles of 292 *Enterococcus* isolates according to antimicrobial class across each sampling time point. Class key: fluoroquinolone = Fluoro, lincosamide = Linco, aminoglycoside = Amino, tetracycline = Tet, macrolide = Macro, nitrofurans = Nitro, penicillin = Pen, streptomycin = Strep, glycylcycline = Glycyl, phenicol = Phenicol.

Among the 292 *Enterococcus* samples, only 9 (3.1%) were pansusceptible to the Gram-positive panel of antimicrobials, and another 33 (11.3%) were resistant to a single antimicrobial, either ciprofloxacin, nitrofurantoin, or lincomycin. Among the 250 Enterococci resistant to more than one antimicrobial, 27 resistance patterns were observed, and 161 (55.1%) of all fecal isolates were resistant to three or more antimicrobials. These resistance patterns most frequently included resistance to tetracyclines, lincosamides, macrolides (erythromycin, tylosin) and streptogramins (Fig. 4). We note that intrinsic resistance to several antimicrobial classes (for example, cephalosporins, lincosamides, aminoglycosides and trimethoprim-sulfamethoxazole) is a recognized feature of enterococci, particularly E. faecium and E. faecalis. Comparison of the resistance patterns across the five *Enterococcus* species is summarized in Table S4.

#### Environmental samples.

No E. coli were isolated from presanitation environmental samples collected prior to placement of the pigs in new barns. In contrast, E. faecium was isolated from presanitation environmental samples from all three treatment rooms before placement of the pigs. In the rooms that would house the Minimal and Moderate treatment groups, E. faecium isolates resistant to two antimicrobials were recovered. Both were resistant to tetracycline, together with resistance to lincomycin (Minimal room) or kanamycin (Moderate room). The E. faecium isolate from the room that would house the Intensive group was resistant to seven of the antimicrobials evaluated (chloramphenicol, erythromycin, lincomycin, streptomycin, quinupristin-dalfopristin, tetracycline, and tylosin). Following sanitation of these rooms prior to pig placement, the environmental samples were negative for both indicator bacterial species. Similarly, neither organism was detected in environmental samples collected at the end of the trial after the facilities were depopulated, sanitized, and disinfected.

## DISCUSSION

This longitudinal study from weaning to market emulated a common clinical scenario (PRRSV challenge) that used antimicrobial treatment protocols that have been employed by practicing veterinarians to treat mild to severe PRRSV associated disease. The timing of the exposures in the nursery phase corresponds with the period in which antimicrobial exposure is most common in pigs, and when the clinical severity of PRRS tends to be greatest. The antimicrobials administered belonged to five medically important classes (tetracycline, macrolide, cephalosporin, penicillins, lincosamide), and were among the most used in the U.S. swine industry (12, 35). However, under the conditions of this study, including substantial quantitative (ATD per 1000 pig days at risk) and qualitative (class, route) differences in antimicrobial exposures between groups, minimal differences in AMR were observed.

Despite a 65.3-fold difference in ATD between groups of pigs, with the exposures concentrated in a 25 day window within the 149 day study period, we found few significant differences between groups, or over time, in the likelihood of phenotypic AMR in commensal bacteria cultured from feces. Furthermore, those significant differences observed could not be easily reconciled with the specific antimicrobials administered or the timing of the exposures of the pigs. The sole difference between groups was for streptomycin in E. coli, yet no aminoglycoside antimicrobials were administered in the study and resistance was higher in the Moderate group than the Minimal and Intensive groups. In all instances where odds of AMR differed between time points, the higher AMR prevalence occurred at the first sampling prior to any antimicrobial exposure. These unanticipated outcomes bear witness to the complexity of use-resistance relationships and have implications for the design and interpretation of research into them.

The foundational premise of antimicrobial stewardship initiatives is that antimicrobial use (AMU) exerts selective pressures which lead to the emergence of resistant pathogens that prejudice the clinical effectiveness of these drugs (36). The mounting crisis of AMR in human medicine bears witness to this intuitive relationship (37, 38), and reduction of use should help to preserve antimicrobial efficacy by reducing selective pressures. However, Knight et al. (2019) state that ‘beyond this empirically well-supported hypothesis…we have not yet convincingly identified the major drivers of the spread of resistance at the population level’ (39). The minimal impacts on AMR observed between groups with substantially different antimicrobial exposures in the current study support this perspective and suggest that clear AMU-AMR patterns may not be reliably demonstrable, particularly in field settings.

It is increasingly recognized that multiple factors other than AMU can have dramatic effects on AMR in populations (40). In food animal production, one potential factor that could influence AMR outcomes is timing of antimicrobial exposure in relation to harvest. There is little published information regarding how the timing of exposure may influence populations of resistant bacteria entering the food supply. With respect to meat industries, exposure of animals to antimicrobials within days of marketing may have very different implications for AMR in the food supply than exposures occurring weeks to months preceding harvest. Results from previous longitudinal field studies of pigs have been highly variable (22, 27). Consistent with the current study, Pollock et al. (2020) reported AMR gene prevalence and abundance were not influenced by antimicrobial exposures (27). This minimal impact of AMU on AMR overall in the current study precluded us from gaining further insight into this question, which has considerable practical importance. It is likely that numerous large studies over the life span of growing pigs will be needed to gain robust understanding of the temporal implications of antimicrobial exposures, and the extent to which results of individual studies should, or should not, be generalized. Important aspects will be to clearly document the sourcing, housing and management of study populations, nutritional history (including the addition of pharmacological levels of zinc and copper in diets), the specific details and timing of antimicrobial exposures, and the choice of outcome variables and criteria for determining resistance. All of these factors have the potential to influence and confound relationships between antimicrobial exposures and AMR.

Another factor that may greatly influence AMR outcomes following antimicrobial exposure is the baseline resistome and microbiome of the study population (41). At the first sampling, phenotypic resistance in E. coli was detected for 11 of the 14 antimicrobials, and in *Enterococcus* for 12 of the 16 antimicrobials. The litters eligible for the study had no history of recent antimicrobial exposure of the sows (i.e., in the current lactation) or piglets, however, it is important to note that the breeding farm was not antimicrobial free. The importance of sows as a source of resistant bacteria to their offspring is self-evident and well established (22, 42). Because this study was conducted in new facilities that had not previously housed animals, the barn environment at placement is not likely to have been a substantial source of bacteria of swine origin. The absence of these organisms in the environmental samples collected before placement of the pigs further supports this hypothesis, and therefore potential confounding due to carryover of resistant organisms from preceding groups of animals or from environmental bacteria was arguably negligible.

The antimicrobials comprising the majority of UDD in this study were tetracyclines, macrolides and cephalosporins, yet no significant differences between groups were found in resistance to these classes. The 504 UDD for tetracyclines (in combination with tiamulin) comprised 53% of UDD in the Intensive group and 42% of UDD across all groups. This metric suggests that the greatest selection pressure from these protocols was likely associated with tetracycline exposure, which occurred over a longer duration (14 days) than did exposures for other classes. Tetracyclines have been widely used over decades in most food animal species globally (9, 43–45) and tetracycline resistance (phenotypically and genotypically) is prevalent in pathogens and commensal bacteria of swine (27, 46–48) and other species, including humans. Tetracycline resistance is also very common in bacteria cultured from environmental samples, and typically comprises a high proportion of genotypic resistance mechanisms within environmental microbiome samples (49, 50). In the current study, tetracycline resistance was widespread across groups and sampling events, including the initial sampling, which may have limited the scope for detecting any increase following exposure to chlortetracycline.

Two additional findings highlighted the complexity of use-resistance relationships. First, exposure to tilmicosin (16-ring macrolide) and ceftiofur (third generation cephalosporin) constituted the majority (96%) of UDD in the Moderate group, and 45% UDD in the Intensive group. Although neither of these classes was administered to the Minimal group, no differences in odds of resistance to cephalosporins or macrolides were observed between any groups. This indicates that negligible selection for resistance to ceftiofur occurred in E. coli under the conditions of this study. We note that ceftiofur was administered intramuscularly and is predominantly excreted in the urine, therefore exposure of intestinal flora to this antimicrobial was likely limited (whereas the macrolide was administered orally). Interestingly, the prevalence of ceftiofur resistance remained stable at around 30% to 40% of E. coli isolated from clinical submissions from diseased swine to the Veterinary Diagnostic Laboratory in Minnesota from 2006 to 2016 (46). This raises questions regarding the temporal dynamics of the emergence of AMR at a broad population level over many years, versus shorter periods of observation as in this study. We note that, unlike the Minimal group, both ceftiofur-exposed groups were also PRRSV challenged, which can affect the pharmacokinetics of ceftiofur, although this effect is mitigated by vaccination (51).

As with all studies, there are strengths and limitations. Strengths included the large differences in antimicrobial exposure between treatment groups, including routes and durations of exposure. Oral administration of antimicrobials is considered more likely than parenteral administration to impact resistance in intestinal bacteria (20, 52, 53). Medication *per os* was included in the two treatment groups (Moderate and Intensive) but not in the control group. Additionally, the Minimal group only received individual treatments when clinically indicated, whereas group treatments of all animals were administered for 5 days (tilmicosin in water) in the Moderate group and more than 20 days (ceftiofur by injection, tilmicosin in water, chlortetracycline/tiamulin in feed) in the Intensive group. Another strength was the range of antimicrobial exposures (ATD per 1000 animal days at risk) used in this study are roughly commensurate with reported ranges administered in swine production in developed countries using analogous measures (10, 11).

Limitations of the study were primarily derived from constraints due to the available facilities and other resources. Most importantly, there was no replication of treatment groups at the group level. The rooms were of identical design, had not previously housed any pigs, and the pigs in each group were matched by litter. These factors should have mitigated potential “group effects,” and the salient finding of the study was the similarity in AMR between the groups rather than their differences. Arguably, the lack of replication at group level would be more problematic when inferring that differences between groups could be causally attributed to the antimicrobial exposures. Second, although steps were taken to minimize risk of transmission between pens, the lack of independence between pens within groups (assumed statistically) could be questioned. Finally, we evaluated only a single isolate of each organism per pen at each sampling event; the use of a single isolate likely underrepresents the diversity of strains and AMR phenotypes present within a typical composite fecal sample. Moreover, although the bacterial species we evaluated are standard indicator organisms used widely in AMR research, they are a miniscule part of the intestinal microbiome. More insight may be gained from an ongoing analysis of the microbiome and resistome in the samples from the study.

In summary, under the conditions of this study, markedly different antimicrobial exposures yielded only minimal differences in AMR patterns over the course of the study. It should be anticipated that outcomes of studies of relationships between antimicrobial use and AMR will be highly heterogeneous due to almost endless permutations of potential antimicrobial exposures (drugs, doses, durations, routes) and methods for quantifying them; the diversity of options for assessing outcomes (samples, organisms/genes, susceptibility testing); the baseline resistance in the study populations and their prior histories of antimicrobial exposures; and the temporal relationships between the exposures and outcomes. This poses enormous challenges to the design and inference of individual studies as well as to the synthesis and interpretation of the collective literature. This study adds to the body of evidence regarding temporal associations between antimicrobial exposures in individual commercial animals and subsequent AMR dynamics in the same animals, with strict control of potential confounding factors. This type of study tends to be underrepresented in the use-resistance association literature, particularly for livestock animals, and thus our results contribute an important additional data point for considering evidence-based guidelines for antimicrobial use, particularly in the context of PRRSV outbreaks.

## MATERIALS AND METHODS

### Study population and design.

The pigs included in the study were selected from a single sow herd in the Midwestern region of the United States that was negative for PRRSV, Influenza A virus, and porcine epidemic diarrhea virus, based on routine clinical and diagnostic monitoring. This source herd was a farrow-to-wean operation housing approximately 5,000 sows. Piglets were weaned at an average of 22.5 days of age. Litters were eligible for inclusion in the study if 1) no cross fostering had been conducted (original litters on sows), 2) the sow had not received an antimicrobial treatment since placement in the farrowing barn, and 3) no piglets in the litter had received antimicrobial treatments. Thirty-six sows of mixed parities that met these criteria were identified. Avoiding low viability piglets, three piglets from each respective litter were selected by convenience and ear tagged. The three pigs from each litter were then randomly assigned across three treatment groups. Thus, a total of 108 weaned pigs (N = 36 per treatment group) comprised the study population.

On the day of weaning, the pigs were commingled in a clean, disinfected, and dried trailer and transported to the research facility. The research facility was a newly constructed barn that had not previously housed any animals prior to placement of the study population. The pigs were weighed on arrival at the research facility then moved into separate rooms by treatment group. In each room, three pigs were placed in each of 12 pens where they remained for the duration of the study. Solid panels were placed between pens to eliminate direct contact and minimize cross-contamination between pens. Each room used negative pressure ventilation with mechanical filters composed of fiberglass media, having a minimum efficiency reported value (MERV) of 16 (Camfil-Farr, Stockholm, Sweden) installed to separately filter all the air entering and exiting each individual room. Additional measures to prevent transmission of microbes between rooms included showering of personnel moving between rooms; an air-purge chamber at the entrances of rooms with pigs; caretakers using a bench to create a transition point to change into room specific boots; and provision of coveralls, gloves, as well as designated equipment and supplies for each room (54).

Pigs in all treatment groups received 2 ml of modified live PRRSV vaccine (Ingelvac PRRS MLV, Boehringer Ingelheim Vetmedica Inc.) 10 days postweaning to reduce severity of clinical illness in the animals later exposed to wild-type PRRSV. One treatment group was not challenged with PRRSV. At 44 days postweaning, the other two groups were inoculated intramuscularly with 2 × 10^3.5^ TCID_50_ of a PRRSV lineage 1 strain 174 field isolate as described previously (33). Clinical assessment of all pigs was conducted by trained personnel using the Individual Pig Care (IPC) scoring system which categorizes pigs according to the perceived severity and duration of clinical signs of any disease they may be experiencing (55, 56). IPC scoring was performed every 3 days for the first 21 days after weaning; three times weekly on Monday, Wednesday, and Friday for 4 weeks after the PRRSV challenge; then once weekly until the end of the trial. Throughout the trial, all individual treatments of pigs were recorded by ear tag number. Mortalities were recorded, including the weights of dead pigs, and all surviving pigs were weighed on the day of marketing (149 days post weaning). All groups were fed the same diets throughout the trial, except for the antimicrobials included in the feed, which varied by treatment group. Pigs were fed eight different rations from postweaning to marketing, which all met or exceeded the National Research Council requirements for swine (57). In line with common industry practices (58), zinc and copper were fed at pharmacological levels in the early nursery rations. Zinc was included at 3000 ppm for the first 11 days on feed, then 2250 ppm from days 12 to 21 on feed. Subsequently zinc was included at 100 ppm to meet nutritional needs. Copper was included at 225 ppm for the first 21 days on feed, then 162 ppm for the remainder of the study.

### Treatment group details.

**FIG 5:**
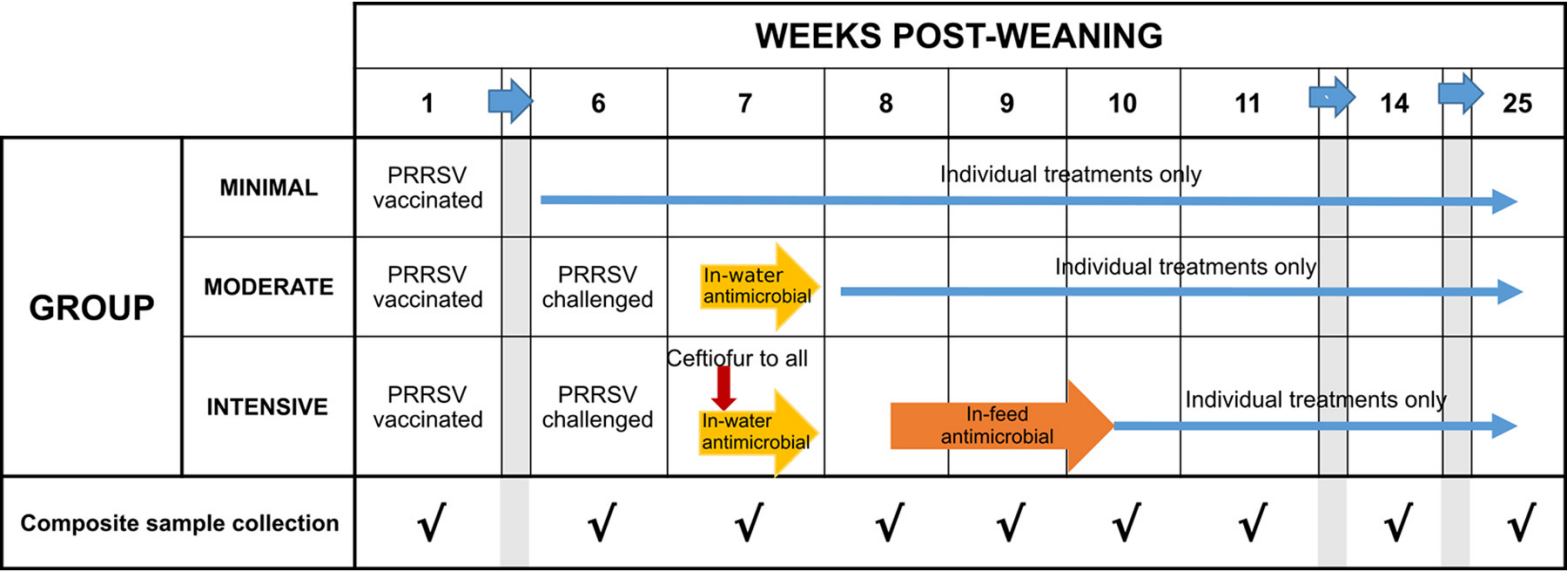
Timeline of PRRS vaccination, PRRSV challenge, group antimicrobial treatments and pen sampling events for Minimal, Moderate, and Intensive treatment groups. When clinically indicated, individual pigs were also treated by injection in all three treatment groups.

The three treatment groups were distinguished by protocols (Minimal, Moderate, Intensive) of antimicrobial use (Fig. 5). In the Minimal Use group, which was not challenged with wild-type PRRSV, antimicrobials (penicillin G procaine (33,000 IU/kg once per day for 3 days) or lincomycin HCl (11 mg/kg once per day for 3 days)) were administered by intramuscular injection to individual pigs for therapeutic purposes when indicated. No antimicrobials were administered in the feed or water. This treatment protocol was also followed for both PRRSV challenged (Moderate and Intensive) groups prior to PRRSV challenge at 44 days post weaning.

In both the Moderate and Intensive groups, starting 5 days post PRRSV challenge (49 days postweaning), tilmicosin phosphate (Pulmotil AC [250 mg/ml], Elanco) was administered in the drinking water via a water medication device, for 5 days (approximately 18.3 mg/kg per day). Subsequently, in the Moderate group, ceftiofur crystalline free acid (EXCEDE for Swine, Zoetis) was administered by intramuscular injection (5 mg/kg once) to individual pigs only when necessary for treatment purposes. No antimicrobials were administered in feed to the Moderate group.

In the Intensive group, in addition to tilmicosin in water, at 7 days postchallenge (51 days postweaning) all pigs received an intramuscular injection of 5 mg/kg ceftiofur crystalline free acid (EXCEDE for Swine, Zoetis). Starting 13 days postchallenge (57 days postweaning), a combination of chlortetracycline (400g/ton to deliver 22 mg/kg BW per day) and tiamulin (35g/ton to deliver 2 mg/kg BW per day) was included in the feed of the Intensive group for 14 days per label requirements. No further antimicrobials were used apart from intramuscular injection of ceftiofur crystalline free acid (5 mg/kg) to one individual pig for treatment purposes.

### Quantification of antimicrobial use.

The overall measure of antimicrobial exposure was Used Daily Doses (UDD), calculated as the total number of animal-days of treatment summed across all active ingredients. This was also expressed in relation to the animal-time at risk as Animal Treatment Days per 1000 animal-days at risk (ATD). The antimicrobial use is summarized in Table 1. A UDD of the combination product of chlortetracycline and tiamulin was defined as 1 day of treatment with the combined product.

**TABLE 1:** Treatment details^a^

Treatment group	Active ingredient	Route	No. of animals treated	Total grams used	Treatment days per regimen	Used daily doses	Animal treatment days (per 1000 days at risk)
Minimal	Penicillin G procaine	Injection	4	2.1	3	12	
	Lincomycin	Injection	1	0.5	3	3	
	Total			**2.6**		**15**	**2.8**
Moderate	Penicillin G procaine	Injection	3	1.5	3	9	
	Ceftiofur	Injection	7	0.9	7	49	
	Tilmicosin	Water	36	112.5	5	180	
	Total			**114.9**		**238**	**44.1**
Intensive	Penicillin G procaine	Injection	1	1.1	3	3	
	Ceftiofur	Injection	36^b^	4.4	7	259	
	Tilmicosin	Water	36	112.5	5	180	
	Chlortetracycline/ tiamulin	Feed	36	460/37.5	14	504	
	Total			**615.5**		**946**	**181.5**

a Used daily doses, total grams (g) used, and animal treatment days per 1000 days at risk were calculated based on actual usage during study. Penicillin G procaine and lincomycin were administered daily for 3 days per treatment regimen; ceftiofur was administered once per treatment regimen.

b Note: 37 individual treatments with ceftiofur were administered to the Intensive treatment group (*n* = 36) as all individuals were treated at 7 days post-PRRSV challenge and one was later also treated individually (11 days post-PRRSV challenge).

### Sample collection, bacterial isolation, and antimicrobial susceptibility testing.

Composite fecal samples were collected from each pen at the following days postweaning: 9, 44, 49, 57, 65, 71, 79, 104 and 149 (Fig. 5). Composite samples were collected using the EZ Reach Sponge Sampler (World Bioproducts, EZ-10NB-CELL-G). One Sponge Sampler was used per pen targeting all evident dunging areas of the pen floor. Environmental samples from the wall surfaces at approximately five feet above the floor (a height which pigs could not contact) and pen floor surfaces (totaling approximately 12 square feet of surface area) were also collected prior to placement of the pigs, and after pigs were marketed (postsanitation).

Samples were transported on ice to an American Association of Veterinary Laboratory Diagnosticians accredited veterinary diagnostic laboratory where they were processed within 24 h after delivery. Formal Standardized Operating Procedures of the laboratory were followed for culture and identification of bacteria, and antimicrobial susceptibility testing. Sponge samples were aseptically placed into sterile plastic bags, mixed with buffered peptone water, and stomached for 2 min at 265 rpm. This rinsate was mixed with double strength MacConkey broth or double strength Enterococcosel broth at a 1:1 ratio (BD Difco manufactured by Becton, Dickinson & Co. Sparks, MD) and incubated at 35°C and 45°C for 24 h for E. coli and *Enterococcus*, respectively. After incubation, a loopful was streaked on Enterococcosel and MacConkey agar (BD Difco manufactured by Becton, Dickinson & Co. Sparks, MD) for *Enterococcus* spp. and E. coli, respectively, then incubated at 35°C for 24 h. For samples with characteristic colonies present on the selective agar plates, one typical well-isolated colony was streaked to blood agar and incubated overnight at 35°C. One colony from the blood agar plates was used for bacterial identity confirmation using Matrix Assisted Laser Desorption Ionization – Time of Flight (MALDI-TOF) technology.

Antimicrobial susceptibility testing was performed using the TREK broth microdilution system (Trek Diagnostic Systems, Cleveland, OH). *Enterococcus* spp. samples were tested with the Sensititre^TM^ Gram Positive NARMS antimicrobial plate (16 antimicrobials plus positive and negative controls). E. coli samples were tested with the Sensititre Gram Negative NARMS antimicrobial plate (14 antimicrobials plus positive and negative controls). Susceptibility breakpoints were adopted from Clinical and Laboratory Standards Institute (CLSI) standards according to NARMS Human Isolate reports (59) as summarized in Table 2. The susceptibility results were dichotomized into resistant or susceptible according to the MIC breakpoints, with intermediate responses being categorized as susceptible. Because CLSI breakpoints have not been established for E. coli for streptomycin or azithromycin, NARMS-established interpretive standards were used as the breakpoints for these antimicrobials (59). Susceptibility results were further analyzed by antimicrobial class.

**TABLE 2:** Antimicrobials tested, antimicrobial class and breakpoint values (μg/ml) for *Enterococcus* spp. and Escherichia coli from pigs

		Breakpoint value (μg/ml)					
Antimicrobial	Antimicrobial	*Enterococcus* spp.			Escherichia coli		
	class	S	I	R	S	I	R
Amoxicillin-clavulanic acid	Penicillin				≤4	8	≥16
Ampicillin	Penicillin				≤8	16	≥32
Azithromycin	Macrolide				NA	N/A	NA
Cefoxitin	Cephalosporin				≤8	16	≥32
Ceftiofur	Cephalosporin				≤2	4	≥8
Ceftriaxone	Cephalosporin				≤1	2	≥4
Chloramphenicol	Amphenicol	≤8	16	≥32	≤8	16	≥32
Ciprofloxacin	Fluoroquinolone	≤1	2	≥4	≤0.06	0.12 to 0.5	≥1
Daptomycin	Cyclic lipopeptide	≤4	NA	≥8			
Erythromycin	Macrolide	≤0.5	1−4	≥8			
Gentamycin	Aminoglycoside	≤500	NA	>500	≤4	8	≥16
Kanamycin	Aminoglycoside	≤512	NA	≥1024			
Lincomycin	Lincosamide	≤2	4	≥8			
Linezolid	Oxazolidinone	≤2	4	≥8			
Nalidixic Acid	Quinolone				≤16	NA	≥32
Nitrofurantoin	Nitrofuran	≤32	64	≥128			
Penicillin	Penicillin	≤8	NA	≥16			
Quinupristin/dalfopristin	Streptogramin	≤1	2	≥4			
Streptomycin	Aminoglycoside	≤1000	NA	>1000	≤32	NA	≥64
Sulfisoxazole	Sulfonamide				≤256	NA	≥512
Tetracycline	Tetracycline	≤4	8	≥16	≤4	8	≥16
Tigecycline	Glycylcycline	≤0.25	NA	≥0.5			
Trimethoprim-sulfamethoxazole	Sulfonamide				≤2	NA	≥4
Tylosin	Macrolide	≤8	16	≥32			
Vancomycin	Glycopeptide	≤4	8−16	≥32			

### Statistical analysis.

For most antimicrobials, only descriptive analysis was reported due to the sparsity of data, i.e., low prevalence of resistance. To analyze potential associations between AMU and AMR in E. coli and *Enterococcus* spp., a generalized estimating equation (GEE) model was generated using SAS software, Version 9 (SAS Institute Inc., Cary, NC, USA). Model effects included treatment group and sample date, alone or in combination, as well as an exchangeable working correlation structure (repeated measure) of pen location within room. Additionally, a binomial distribution and logit function were specified to reflect the dichotomous nature of the outcome variable (i.e., susceptible/resistant). The primary outcome was the antimicrobial susceptibility status of each isolate to the antimicrobial of interest. In initial model evaluations, only three model including a treatment group by sample-date interaction term converged and the Quasi Information Criterion (QIC) indicated that these were not the preferred models. The QIC criteria indicated that the most parsimonious models were those that did not include sample date for most bacteria-antimicrobial combinations. However, as analysis of the temporal effects of the antimicrobial exposure was a primary goal of the study, the final model selected included both treatment group and sample date as well as the repeated measures factor. Model comparison data are presented in Table S5. For antimicrobials for which resistance was relatively common and the model converged, odds ratio estimates were calculated with 95% confidence intervals for sample date (including erythromycin, quinupristin-dalfopristin, and tylosin for *Enterococcus* isolates) and treatment group (including streptomycin for E. coli) comparisons. Statistical significance was indicated at *P* < 0.05 after adjusting for multiple comparisons using the Tukey-Kramer method. Prevalence of resistant isolates was compared between treatments, with statistical significance indicated at *P* < 0.05. One-way ANOVA was used to compare average daily gain between treatment groups and a two-sample *t* test was conducted to compare average daily gain between PRRSV challenged versus nonchallenged groups (Statistix 10.0. Analytical Software, Tallahassee, FL, USA).
