# Supplementary material for: Evaluation of the Impact of Antimicrobial Use Protocols in Porcine Reproductive and Respiratory Syndrome Virus-Infected Swine on Phenotypic Antimicrobial Resistance Patterns
Source: Appl Environ Microbiol. 2022 Jan 11;88(1):e00970-21. doi: 10.1128/AEM.00970-21 (PMC8752131; doi:10.1128/AEM.00970-21)
Supplement: Supplemental file 1 — Tables S1 to S5. Download AEM.00970-21-s0001.pdf, PDF file, 0.2 MB [file aem.00970-21-s0001.pdf]

	Antimicrobial agent	% AMR	<=0.015	=0.03	=0.06	<=0.12	=0.12	<=0.25	=0.25	<=0.5	=0.5	<=1	=1	=2	<=4	=4	> 4	=8	>8	<=16	=16	>16	=32	>32	=64	>64	>256	Total	Range of Dilution tested
Minimal Group	Amox / Clav	3.8												29		44		28					3	1				105	1/0.5 - 32/16
	Amp	28.6										14		38		23								30				105	1-32
	Azith	0.0											1	23		76		5					Rǂ	Rǂ				105	0.12 - 16*
	Cefox	3.8												28		69		4						4				105	0.5 - 32
	Ceftio	3.8							32		66		3					1	3									105	0.12 - 8
	Ceftria	3.8						100			1										3		1					105	0.25 - 64
	Chlor	8.6														25		69			2		1	8				105	2-32
	Cipro	0.0	98	4					2		1																	105	0.015 - 4
	Genta	1.0									42		58	4								1						105	0.25 - 16
	Nalid	1.0											10	77		15		2						1				105	0.5 - 32
	Strep	23.8														11		45			16		8		10	15		105	2-64.
	Sulfisox	0.0																		62			25		2		16	105	16 - 256**
	Tetra	83.8													17								9	79				105	4-32
	Trim / Sulfa	0.0				92			11		2																	105	0.12/2.4 - 4/76
Moderate Group	Amox / Clav	1.0										1		8		46		45			1							101	1/0.5 - 32/16
	Amp	47.5										4		24		24					1		1	47				101	1-32
	Azith	0.0												24		73		4					Rǂ	Rǂ				101	0.12 - 16*
	Cefox	0.0								1				9		78		13										101	0.5 - 32
	Ceftio	0.0				1			23		76		1															101	0.12 - 8
	Ceftria	0.0						101																				101	0.25 - 64
	Chlor	3.0														29		65			4		1	2				101	2-32
	Cipro	0.0	97	3							1																	101	0.015 - 4
	Genta	1.0						5			30		61	4								1						101	0.25 - 16
	Nalid	0.0											9	64		27					1							101	0.5 - 32
	Strep	60.4														4		25			7		4		25	36		101	2-64.
	Sulfisox	0.0																		59			14		18		10	101	16 - 256**
	Tetra	91.1													9								1	91				101	4-32
	Trim / Sulfa	0.0				91			7		3																	101	0.12/2.4 - 4/76
Intensive Group	Amox / Clav	3.0										1		23		32		42			2			1				101	1/0.5 - 32/16
	Amp	35.6										10		22		28		4			1			36				101	1-32
	Azith	0.0											2	16		64		18				1	Rǂ	1				102	0.12 - 16*
	Cefox	1.0											3	16		73		7			1			1				101	0.5 - 32
	Ceftio	1.0				3			29		63		5						1									101	0.12 - 8
	Ceftria	1.0						99			1										1							101	0.25 - 64
	Chlor	8.9														25		64			3		4	5				101	2-32
	Cipro	1.0	89		2		1		1		7						1											101	0.015 - 4
	Genta	2.0									36		57	5		1					1	1						101	0.25 - 16
	Nalid	4.0											16	68		7					6		1	3				101	0.5 - 32
	Strep	21.8														5		48			17		9		9	13		101	2-64.
	Sulfisox	0.0																		71			15				15	101	16 - 256**
	Tetra	93.1													7								5	89				101	4-32
	Trim / Sulfa	6.9				87			3		3		1				7											101	0.12/2.4 - 4/76

Table S1. Distribution (number of isolates) of minimum inhibitory concentrations (MICs) for *Escherichia coli* in swine across all sampling events. Rǂ: CLSI breakpoints are not established for azithromycin and E. coli; interpretive standards used are NARMS-established breakpoints

for resistance monitoring and should not be used to predict clinical efficacy. *: Final dilution tested was >16 corresponding with ≥32. **: Final dilution tested was >256 corresponding with ≥512.

Group	Antimicrobial agent	% AMR	=0.03	=0.06	=0.12	<=0.25	=0.25	<=0.5	=0.5	<=1	=1	=2	=4	>4	=8	>8	=16	=32	>32	=64	>64	<=128	256	<=512	512	1024	>1024	=2048	>2048	Total	Range of dilution tested
Minimal Group	Chlor	0.0											6		87		7													100	2-32
	Cipro	17.0					1		31		23	28	12	5																100	0.12 - 4
	Dapto	5.0									14	41	40		5															100	0.25 - 16
	Eryth	44.0				19					12	18	7		2	42														100	0.25 - 8
	Genta	1.0																				98	2							100	128 - 1024
	Kanam	8.0																				83	7		2		8			100	128 - 1024
	Linco	96.0								4					1	95														100	1-8
	Linez	0.0										92	8																	100	0.5 - 8
	Nitro	4.0													2		17	22		55	4									100	2-64
	Pen	1.0									10	22	59		8		1													100	0.25 - 16
	Quin/Dalf	55.0						4			1	40	21		11		13	7	3											100	0.5 - 32
	Strep	9.0																						91		4		4	1	100	512 - 2048
	Tetra	72.0								28									72											100	1-32
	Tige	1.0	5	27	53		14		1																					100	0.015 - 0.5
	Tylosin	41.0									1	16	15		23		4		41											100	0.25 - 32
	Vanco	0.0							57		33	10																		100	0.25 - 32
Moderate Group	Chlor	1.1											9		73		9	1												92	2-32
	Cipro	6.5					2		37		23	24	5	1																92	0.12 - 4
	Dapto	4.3									20	40	28		4															92	0.25 - 16
	Eryth	47.8				31					3	6	8		2	42														92	0.25 - 8
	Genta	3.3																				87	4				1			92	128 - 1024
	Kanam	17.4																				67	7		2		16			92	128 - 1024
	Linco	93.5								3		3				86														92	1-8
	Linez	0.0									5	81	6																	92	0.5 - 8
	Nitro	11.9													2		18	31		30	11									92	2-64
	Pen	1.1				1					14	30	44		2		1													92	0.25 - 16
	Quin/Dalf	50.0						3			2	41	16		13		5	10	2											92	0.5 - 32
	Strep	17.4																						76		6		6	4	92	512 - 2048
	Tetra	72.8								24		1							67											92	1-32
	Tige	0.0	4	25	53		10																							92	0.015 - 0.5
	Tylosin	44.6									1	3	31		14		2		41											92	0.25 - 32
	Vanco	0.0							58		33	1																		92	0.25 - 32
Intensive Group	Chlor	0.0											26		73		1													100	2-32
	Cipro	6.0							53		29	12	3	3																100	0.12 - 4
	Dapto	1.0									29	51	19		1															100	0.25 - 16
	Eryth	48.0				33			1		5	7	6			48														100	0.25 - 8
	Genta	6.0																				93	1		3	2	1			100	128 - 1024
	Kanam	12.0																				87	1				12			100	128 - 1024
	Linco	94.0								5		1			1	93														100	1-8
	Linez	0.0									4	96																		100	0.5 - 8
	Nitro	4.0															18	47		31	4									100	2-64
	Pen	5.0				1					24	40	28		2		5													100	0.25 - 16
	Quin/Dalf	51.0						5			2	42	15		21		10	5												100	0.5 - 32
	Strep	14.0																						86		4		5	5	100	512 - 2048
	Tetra	85.0								15								1	84											100	1-32
	Tige	0.0	2	27	60		11																							100	0.015 - 0.5
	Tylosin	49.0									3	12	30		6				49											100	0.25 - 32
	Vanco	0.0							72		24	4																		100	0.25 - 32

Table S2. Distribution (number of isolates) of minimum inhibitory concentrations (MICs) for *Enterococcus sp.* in swine across all sampling events.

Bacteria	Antimicrobial	Group	Week	N	Proportion Resistant	Lower CI	Upper CI
E. coli	Amoxicillin / Clavulonic Acid	Min	1	12	16.6%	2.0%	48.4%
		Min	6	11	9.0%	0.2%	41.2%
		Min	7	12	0%	0%	26.5%
		Min	8	12	0%	0%	26.5%
		Min	9	11	9.0%	0.2%	41.2%
		Min	10	12	0%	0%	26.5%
		Min	11	11	0%	0%	28.5%
		Min	14	12	0%	0%	26.5%
		Min	25	12	0%	0%	26.5%
		Mod	1	12	0%	0%	26.5%
		Mod	6	9	0%	0%	33.6%
		Mod	7	12	0%	0%	26.5%
		Mod	8	11	0%	0%	28.5%
		Mod	9	11	0%	0%	28.5%
		Mod	10	12	0%	0%	26.5%
		Mod	11	10	0%	0%	30.8%
		Mod	14	12	0%	0%	26.5%
		Mod	25	12	8.3%	0.2%	38.4%
		Int	1	9	11.1%	0.2%	48.2%
		Int	6	11	9.0%	0.2%	41.2%
		Int	7	11	0%	0%	28.5%
		Int	8	11	0%	0%	28.5%
		Int	9	12	0%	0%	26.5%
		Int	10	12	0%	0%	26.5%
		Int	11	11	0%	0%	28.5%
		Int	14	12	0%	0%	26.5%
		Int	25	12	8.3%	0.2%	38.4%
E. coli	Ampicillin	Min	1	12	33.3%	9.9%	65.1%
		Min	6	11	36.3%	10.9%	69.2%
		Min	7	12	41.6%	15.1%	72.3%
		Min	8	12	41.6%	15.1%	72.3%
		Min	9	11	18.1%	2.2%	51.7%
		Min	10	12	25.0%	5.4%	57.1%
		Min	11	11	27.2%	6.0%	60.9%
		Min	14	12	16.6%	2.0%	48.4%
		Min	25	12	16.6%	2.0%	48.4%
		Mod	1	12	41.6%	15.1%	72.3%
		Mod	6	9	55.5%	21.2%	86.3%
		Mod	7	12	58.3%	27.6%	84.8%
		Mod	8	11	45.4%	16.7%	76.6%
		Mod	9	11	54.5%	23.3%	83.2%
		Mod	10	12	58.3%	27.6%	84.8%
		Mod	11	10	50.0%	18.7%	81.2%
		Mod	14	12	41.6%	15.1%	72.3%

E. coli	Ampicillin (continued)	Mod	25	12	25.0%	5.4%	5.7%
		Int	1	9	44.4%	13.7%	78.8%
		Int	6	11	36.3%	10.9%	69.2%
		Int	7	11	45.4%	16.7%	76.6%
		Int	8	11	18.1%	2.2%	51.7%
		Int	9	12	8.3%	0.2%	38.4%
		Int	10	12	50.0%	21.0%	78.9%
		Int	11	11	36.3%	10.9%	69.2%
		Int	14	12	33.3%	9.9%	65.1%
		Int	25	12	50.0%	21.0%	78.9%
E. coli	Azithromycin	Min	1	12	0%	0%	26.5%
		Min	6	11	0%	0%	28.5%
		Min	7	12	0%	0%	26.5%
		Min	8	12	0%	0%	26.5%
		Min	9	11	0%	0%	28.5%
		Min	10	12	0%	0%	26.5%
		Min	11	11	0%	0%	28.5%
		Min	14	12	0%	0%	26.5%
		Min	25	12	0%	0%	26.5%
		Mod	1	12	0%	0%	26.5%
		Mod	6	9	0%	0%	33.6%
		Mod	7	12	0%	0%	26.5%
		Mod	8	11	0%	0%	28.5%
		Mod	9	11	0%	0%	28.5%
		Mod	10	12	0%	0%	26.5%
		Mod	11	10	0%	0%	30.8%
		Mod	14	12	0%	0%	26.5%
		Mod	25	12	0%	0%	26.5%
		Int	1	9	0%	0%	33.6%
		Int	6	11	0%	0%	28.5%
		Int	7	11	0%	0%	28.5%
		Int	8	11	9.0%	0.2%	41.2%
		Int	9	12	0%	0%	26.5%
		Int	10	12	0%	0%	26.5%
		Int	11	11	0%	0%	28.5%
		Int	14	12	0%	0%	26.5%
		Int	25	12	0%	0%	26.5%
E. coli	Cefoxitin	Min	1	12	16.6%	2.0%	48.4%
		Min	6	11	9.0%	0.2%	41.2%
		Min	7	12	0%	0%	26.5%
		Min	8	12	0%	0%	26.5%
		Min	9	11	9.0%	0.2%	41.2%
		Min	10	12	0%	0%	26.5%
		Min	11	11	0%	0%	28.5%
		Min	14	12	0%	0%	26.5%
		Min	25	12	0%	0%	26.5%
		Mod	1	12	0%	0%	26.5%

E. coli	Cefoxitin (continued)	Mod	6	9	0%	0%	33.6%
		Mod	7	12	0%	0%	26.5%
		Mod	8	11	0%	0%	28.5%
		Mod	9	11	0%	0%	28.5%
		Mod	10	12	0%	0%	26.5%
		Mod	11	10	0%	0%	30.8%
		Mod	14	12	0%	0%	26.5%
		Mod	25	12	0%	0%	26.5%
		Int	1	9	0%	0%	33.6%
		Int	6	11	0%	0%	28.5%
		Int	7	11	0%	0%	28.5%
		Int	8	11	0%	0%	28.5%
		Int	9	12	0%	0%	26.5%
		Int	10	12	0%	0%	26.5%
		Int	11	11	0%	0%	28.5%
		Int	14	12	0%	0%	26.5%
		Int	25	12	8.3%	0.2%	38.4%
E. coli	Ceftiofur	Min	1	12	16.6%	2.0%	48.4%
		Min	6	11	9.0%	0.2%	41.2%
		Min	7	12	0%	0%	26.5%
		Min	8	12	0%	0%	26.5%
		Min	9	11	9.0%	0.2%	41.2%
		Min	10	12	0%	0%	26.5%
		Min	11	11	0%	0%	28.5%
		Min	14	12	0%	0%	26.5%
		Min	25	12	0%	0%	26.5%
		Mod	1	12	0%	0%	26.5%
		Mod	6	9	0%	0%	33.6%
		Mod	7	12	0%	0%	26.5%
		Mod	8	11	0%	0%	28.5%
		Mod	9	11	0%	0%	28.5%
		Mod	10	12	0%	0%	26.5%
		Mod	11	10	0%	0%	30.8%
		Mod	14	12	0%	0%	26.5%
		Mod	25	12	0%	0%	26.5%
		Int	1	9	0%	0%	33.6%
		Int	6	11	0%	0%	28.5%
		Int	7	11	0%	0%	28.5%
		Int	8	11	0%	0%	28.5%
		Int	9	12	0%	0%	26.5%
		Int	10	12	0%	0%	26.5%
		Int	11	11	0%	0%	28.5%
		Int	14	12	0%	0%	26.5%
		Int	25	12	8.3%	0.2%	38.4%
E. coli	Ceftriaxone	Min	1	12	16.6%	2.0%	48.4%
		Min	6	11	9.0%	0.2%	41.2%
		Min	7	12	0%	0%	26.5%

E. coli	Ceftriaxone (continued)	Min	8	12	0%	0%	26.5%
		Min	9	11	9.0%	0.2%	41.2%
		Min	10	12	0%	0%	26.5%
		Min	11	11	0%	0%	28.5%
		Min	14	12	0%	0%	26.5%
		Min	25	12	0%	0%	26.5%
		Mod	1	12	0%	0%	26.5%
		Mod	6	9	0%	0%	33.6%
		Mod	7	12	0%	0%	26.5%
		Mod	8	11	0%	0%	28.5%
		Mod	9	11	0%	0%	28.5%
		Mod	10	12	0%	0%	26.5%
		Mod	11	10	0%	0%	30.8%
		Mod	14	12	0%	0%	26.5%
		Mod	25	12	0%	0%	26.5%
		Int	1	9	0%	0%	33.6%
		Int	6	11	0%	0%	28.5%
		Int	7	11	0%	0%	28.5%
		Int	8	11	0%	0%	28.5%
		Int	9	12	0%	0%	26.5%
		Int	10	12	0%	0%	26.5%
		Int	11	11	0%	0%	28.5%
		Int	14	12	0%	0%	26.5%
		Int	25	12	0%	0%	26.5%
E. coli	Chloramphenicol	Min	1	12	8.3%	0.2%	38.4%
		Min	6	11	9.0%	0.2%	41.2%
		Min	7	12	8.3%	0.2%	38.4%
		Min	8	12	8.3%	0.2%	38.4%
		Min	9	11	0%	0%	28.5%
		Min	10	12	0%	0%	26.5%
		Min	11	11	9.0%	0.2%	41.2%
		Min	14	12	16.6%	2.0%	48.4%
		Min	25	12	16.6%	2.0%	48.4%
		Mod	1	12	0%	0%	26.5%
		Mod	6	9	0%	0%	33.6%
		Mod	7	12	0%	0%	26.5%
		Mod	8	11	9.0%	0.2%	41.2%
		Mod	9	11	0%	0%	28.5%
		Mod	10	12	8.3%	0.2%	38.4%
		Mod	11	10	0%	0%	30.8%
		Mod	14	12	8.3%	0.2%	38.4%
		Mod	25	12	0%	0%	26.5%
		Int	1	9	11.0%	0.2%	48.2%
		Int	6	11	0%	0%	28.5%
		Int	7	11	0%	0%	28.5%
		Int	8	11	0%	0%	28.5%
		Int	9	12	16.6%	2.0%	48.4%

E. coli	Chloramphenicol (continued)	Int	10	12	8.3%	0.2%	38.4%
		Int	11	11	9.0%	0.2%	41.2%
		Int	14	12	16.6%	2.0%	48.4%
		Int	25	12	16.6%	2.0%	48.4%
E. coli	Ciprofloxacin	Min	1	12	0%	0%	26.5%
		Min	6	11	0%	0%	28.5%
		Min	7	12	0%	0%	26.5%
		Min	8	12	0%	0%	26.5%
		Min	9	11	0%	0%	28.5%
		Min	10	12	0%	0%	26.5%
		Min	11	11	0%	0%	28.5%
		Min	14	12	0%	0%	26.5%
		Min	25	12	0%	0%	26.5%
		Mod	1	12	0%	0%	26.5%
		Mod	6	9	0%	0%	33.6%
		Mod	7	12	0%	0%	26.5%
		Mod	8	11	0%	0%	28.5%
		Mod	9	11	0%	0%	28.5%
		Mod	10	12	0%	0%	26.5%
		Mod	11	10	0%	0%	30.8%
		Mod	14	12	0%	0%	26.5%
		Mod	25	12	0%	0%	26.5%
		Int	1	9	0%	0%	33.6%
		Int	6	11	0%	0%	28.5%
		Int	7	11	9.0%	0.2%	41.2%
		Int	8	11	0%	0%	28.5%
		Int	9	12	0%	0%	26.5%
		Int	10	12	0%	0%	26.5%
		Int	11	11	0%	0%	28.5%
		Int	14	12	0%	0%	26.5%
		Int	25	12	0%	0%	26.5%
E. coli	Gentamicin	Min	1	12	8.3%	0.2%	38.4%
		Min	6	11	0%	0%	28.5%
		Min	7	12	0%	0%	26.5%
		Min	8	12	0%	0%	26.5%
		Min	9	11	0%	0%	28.5%
		Min	10	12	0%	0%	26.5%
		Min	11	11	0%	0%	28.5%
		Min	14	12	0%	0%	26.5%
		Min	25	12	0%	0%	26.5%
		Mod	1	12	0%	0%	26.5%
		Mod	6	9	0%	0%	33.6%
		Mod	7	12	8.3%	0.2%	38.4%
		Mod	8	11	0%	0%	28.5%
		Mod	9	11	0%	0%	28.5%
		Mod	10	12	0%	0%	26.5%
		Mod	11	10	0%	0%	30.8%

E. coli	Gentamicin (continued)	Mod	14	12	0%	0%	26.5%
		Mod	25	12	0%	0%	26.5%
		Int	1	9	0%	0%	33.6%
		Int	6	11	0%	0%	28.5%
		Int	7	11	9.0%	0.2%	41.2%
		Int	8	11	0%	0%	28.5%
		Int	9	12	0%	0%	26.5%
		Int	10	12	0%	0%	26.5%
		Int	11	11	0%	0%	28.5%
		Int	14	12	0%	0%	26.5%
		Int	25	12	8.3%	0.2%	38.4%
E. coli	Nalidixic Acid	Min	1	12	8.3%	0.2%	38.4%
		Min	6	11	0%	0%	28.5%
		Min	7	12	0%	0%	26.5%
		Min	8	12	0%	0%	26.5%
		Min	9	11	0%	0%	28.5%
		Min	10	12	0%	0%	26.5%
		Min	11	11	0%	0%	28.5%
		Min	14	12	0%	0%	26.5%
		Min	25	12	0%	0%	26.5%
		Mod	1	12	0%	0%	26.5%
		Mod	6	9	0%	0%	33.6%
		Mod	7	12	0%	0%	26.5%
		Mod	8	11	0%	0%	28.5%
		Mod	9	11	0%	0%	28.5%
		Mod	10	12	0%	0%	26.5%
		Mod	11	10	0%	0%	30.8%
		Mod	14	12	0%	0%	26.5%
		Mod	25	12	0%	0%	26.5%
		Int	1	9	0%	0%	33.6%
		Int	6	11	9.0%	0.2%	41.2%
		Int	7	11	9.0%	0.2%	41.2%
		Int	8	11	0%	0%	28.5%
		Int	9	12	0%	0%	26.5%
		Int	10	12	0%	0%	26.5%
		Int	11	11	9.0%	0.2%	41.2%
		Int	14	12	8.3%	0.2%	38.4%
		Int	25	12	0%	0%	26.5%
E. coli	Streptomycin	Min	1	12	50.0%	21.0%	78.9%
		Min	6	11	18.1%	2.2%	51.7%
		Min	7	12	25.0%	5.4%	57.1%
		Min	8	12	25.0%	5.4%	57.1%
		Min	9	11	0.0%	0%	28.5%
		Min	10	12	16.6%	2.0%	48.4%
		Min	11	11	27.2%	6.0%	60.9%
		Min	14	12	25.0%	5.4%	57.1%
		Min	25	12	25.0%	5.4%	57.1%

E. coli	Streptomycin (continued)	Mod	1	12	66.6%	34.8%	90.0%
		Mod	6	9	55.5%	21.2%	86.3%
		Mod	7	12	66.6%	34.8%	90.0%
		Mod	8	11	63.6%	30.7%	89.0%
		Mod	9	11	72.7%	39.0%	93.9%
		Mod	10	12	58.3%	27.6%	84.8%
		Mod	11	10	60.0%	26.2%	87.8%
		Mod	14	12	66.6%	34.8%	90.0%
		Mod	25	12	33.3%	9.9%	65.1%
		Int	1	9	44.4%	13.7%	78.8%
		Int	6	11	45.4%	16.7%	76.6%
		Int	7	11	36.3%	10.9%	69.2%
		Int	8	11	27.2%	6.0%	60.9%
		Int	9	12	25.0%	5.4%	57.1%
		Int	10	12	8.3%	0.2%	38.4%
		Int	11	11	18.1%	2.2%	51.7%
		Int	14	12	0%	0%	26.5%
		Int	25	12	0%	0%	26.5%
E. coli	Sulfisoxazole	Min	1	12	16.6%	2.0%	48.4%
		Min	6	11	27.2%	6.0%	60.9%
		Min	7	12	8.3%	0.2%	38.4%
		Min	8	12	33.3%	9.9%	65.1%
		Min	9	11	0%	0%	28.5%
		Min	10	12	0%	0%	26.5%
		Min	11	11	18.1%	2.2%	51.7%
		Min	14	12	16.6%	2.0%	48.4%
		Min	25	12	16.6%	2.0%	48.4%
		Mod	1	12	8.3%	0.2%	38.4%
		Mod	6	9	22.2%	2.8%	60.0%
		Mod	7	12	8.3%	0.2%	38.4%
		Mod	8	11	18.1%	2.0%	51.7%
		Mod	9	11	9.0%	0.2%	41.2%
		Mod	10	12	16.6%	2.0%	48.4%
		Mod	11	10	0%	0%	30.8%
		Mod	14	12	8.3%	0.2%	38.4%
		Mod	25	12	0%	0%	26.5%
		Int	1	9	11.1%	0.3%	48.2%
		Int	6	11	18.1%	2.2%	51.7%
		Int	7	11	9.0%	0.2%	41.2%
		Int	8	11	9.0%	0.2%	41.2%
		Int	9	12	25.0%	5.4%	57.1%
		Int	10	12	16.6%	2.0%	48.4%
		Int	11	11	9.0%	0.2%	41.2%
		Int	14	12	16.6%	2.0%	48.4%
		Int	25	12	16.6%	2.0%	48.4%
E. coli	Tetracycline	Min	1	12	83.3%	51.5%	97.9%
		Min	6	11	81.8%	48.2%	97.7%

E. coli	Tetracycline (continued)	Min	7	12	83.3%	51.5%	97.9%
		Min	8	12	75.0%	42.8%	94.5%
		Min	9	11	72.7%	39.0%	93.9%
		Min	10	12	91.6%	61.5%	99.7%
		Min	11	11	90.9%	58.7%	99.7%
		Min	14	12	83.3%	51.5%	97.9%
		Min	25	12	91.6%	61.5%	99.7%
		Mod	1	12	91.6%	61.5%	99.7%
		Mod	6	9	100%	66.3%	100%
		Mod	7	12	91.6%	61.5%	99.7%
		Mod	8	11	100%	71.5%	100%
		Mod	9	11	81.8%	48.2%	97.7%
		Mod	10	12	91.6%	61.5%	99.7%
		Mod	11	10	90.0%	55.5%	99.7%
		Mod	14	12	91.6%	61.5%	99.7%
		Mod	25	12	83.3%	51.5%	97.9%
		Int	1	9	100%	66.3%	100%
		Int	6	11	81.8%	48.2%	97.7%
		Int	7	11	90.9%	58.7%	99.7%
		Int	8	11	81.8%	48.2%	97.7%
		Int	9	12	100%	73.5%	100%
		Int	10	12	100%	73.5%	100%
		Int	11	11	90.9%	58.7%	99.7%
		Int	14	12	91.6%	61.5%	99.7%
		Int	25	12	100%	73.5%	100%
E. coli	Trimethoprim/Sulfa	Min	1	12	0%	0%	26.5%
		Min	6	11	0%	0%	28.5%
		Min	7	12	0%	0%	26.5%
		Min	8	12	0%	0%	26.5%
		Min	9	11	0%	0%	28.5%
		Min	10	12	0%	0%	26.5%
		Min	11	11	0%	0%	28.5%
		Min	14	12	0%	0%	26.5%
		Min	25	12	0%	0%	26.5%
		Mod	1	12	0%	0%	26.5%
		Mod	6	9	0%	0%	33.6%
		Mod	7	12	0%	0%	26.5%
		Mod	8	11	0%	0%	28.5%
		Mod	9	11	0%	0%	28.5%
		Mod	10	12	0%	0%	26.5%
		Mod	11	10	0%	0%	30.8%
		Mod	14	12	0%	0%	26.5%
		Mod	25	12	0%	0%	26.5%
		Int	1	9	0%	0%	33.6%
		Int	6	11	0%	0%	28.5%
		Int	7	11	0%	0%	28.5%
		Int	8	11	0%	0%	28.5%

E. coli	Trimethoprim/Sulfa (continued)	Int	9	12	16.6%	2.0%	48.4%
		Int	10	12	8.3%	0.2%	38.4%
		Int	11	11	9.0%	0.2%	41.2%
		Int	14	12	16.6%	2.0%	48.4%
		Int	25	12	8.3%	0.2%	38.4%
Enterococcus	Chloramphenicol	Min	1	12	0%	0%	26.5%
		Min	6	10	0%	0%	30.8%
		Min	7	12	0%	0%	26.5%
		Min	8	7	0%	0%	41.0%
		Min	9	12	0%	0%	26.5%
		Min	10	12	0%	0%	26.5%
		Min	11	11	0%	0%	28.5%
		Min	14	12	0%	0%	26.5%
		Min	25	12	0%	0%	26.5%
		Mod	1	12	0%	0%	26.5%
		Mod	6	12	0%	0%	26.5%
		Mod	7	10	0%	0%	30.8%
		Mod	8	8	0%	0%	36.9%
		Mod	9	10	0%	0%	30.8%
		Mod	10	9	0%	0%	33.6%
		Mod	11	10	10.0%	0.2%	44.5%
		Mod	14	9	0%	0%	33.6%
		Mod	25	11	0%	0%	28.5%
		Int	1	12	0%	0%	26.5%
		Int	6	11	0%	0%	28.5%
		Int	7	9	0%	0%	33.6%
		Int	8	12	0%	0%	26.5%
		Int	9	11	0%	0%	28.5%
		Int	10	11	0%	0%	28.5%
		Int	11	12	0%	0%	26.5%
		Int	14	12	0%	0%	26.5%
		Int	25	11	0%	0%	28.5%
Enterococcus	Ciprofloxacin	Min	1	12	8.3%	0.2%	38.4%
		Min	6	10	50.0%	18.7%	81.2%
		Min	7	12	50.0%	21.0%	78.9%
		Min	8	7	14.2%	0.3%	57.8%
		Min	9	12	16.6%	2.0%	48.4%
		Min	10	12	0%	0%	26.5%
		Min	11	11	18.1%	2.2%	51.7%
		Min	14	12	0%	0%	26.5%
		Min	25	12	0%	0%	26.5%
		Mod	1	12	16.6%	2.0%	48.4%
		Mod	6	12	8.3%	0.2%	38.4%
		Mod	7	10	10.0%	0.2%	44.5%
		Mod	8	8	0%	0%	36.9%
		Mod	9	10	10.0%	0.2%	44.5%
		Mod	10	9	0%	0%	33.6%

Enterococcus	Ciprofloxacin (continued)	Mod	11	10	10.0%	0.2%	44.5%
		Mod	14	9	0%	0%	33.6%
		Mod	25	11	0%	0%	28.5%
		Int	1	12	0%	0%	26.5%
		Int	6	11	9.0%	0.2%	41.2%
		Int	7	9	0%	0%	33.6%
		Int	8	12	0%	0%	26.5%
		Int	9	11	0%	0%	28.5%
		Int	10	11	0%	0%	28.5%
		Int	11	12	8.3%	0.2%	38.4%
		Int	14	12	16.6%	2.0%	48.4%
		Int	25	11	18.1%	2.0%	51.7%
Enterococcus	Daptomycin	Min	1	12	0%	0%	26.5%
		Min	6	10	0%	0%	30.8%
		Min	7	12	0%	0%	26.5%
		Min	8	7	14.2%	0.3%	57.8%
		Min	9	12	16.6%	2.0%	48.4%
		Min	10	12	8.3%	0.2%	38.4%
		Min	11	11	0%	0%	28.5%
		Min	14	12	8.3%	0.2%	38.4%
		Min	25	12	0%	0%	26.5%
		Mod	1	12	8.3%	0.2%	38.4%
		Mod	6	12	0%	0%	26.5%
		Mod	7	10	0%	0%	30.8%
		Mod	8	8	0%	0%	36.9%
		Mod	9	10	0%	0%	30.8%
		Mod	10	9	11.1%	0.2%	48.2%
		Mod	11	10	0%	0%	30.8%
		Mod	14	9	11.1%	0.2%	48.2%
		Mod	25	11	9.0%	0.2%	41.2%
		Int	1	12	0%	0%	26.5%
		Int	6	11	0%	0%	28.5%
		Int	7	9	0%	0%	33.6%
		Int	8	12	0%	0%	26.5%
		Int	9	11	0%	0%	28.5%
		Int	10	11	0%	0%	28.5%
		Int	11	12	0%	0%	26.5%
		Int	14	12	8.3%	0.2%	38.4%
		Int	25	11	0%	0%	28.5%
Enterococcus	Erythromycin	Min	1	12	91.6%	61.5%	99.7%
		Min	6	10	30.0%	6.6%	65.2%
		Min	7	12	16.6%	2.0%	48.4%
		Min	8	7	0.0%	0%	41.0%
		Min	9	12	33.0%	9.9%	65.1%
		Min	10	12	50.0%	21.0%	78.9%
		Min	11	11	54.5%	23.3%	83.2%
		Min	14	12	50.0%	21.0%	78.9%

Enterococcus	Erythromycin (continued)	Min	25	12	50.0%	21.0%	78.9%
		Mod	1	12	66.6%	34.8%	90.0%
		Mod	6	12	41.6%	15.1%	72.3%
		Mod	7	10	10.0%	0.2%	44.5%
		Mod	8	8	50.0%	15.7%	84.3%
		Mod	9	10	50.0%	18.7%	81.2%
		Mod	10	9	33.3%	7.4%	70.0%
		Mod	11	10	70.0%	34.7%	93.3%
		Mod	14	9	77.7%	39.9%	97.1%
		Mod	25	11	36.3%	10.9%	69.2%
		Int	1	12	66.6%	34.8%	90.0%
		Int	6	11	27.2%	6.0%	60.9%
		Int	7	9	44.4%	13.7%	78.8%
		Int	8	12	58.3%	27.6%	84.8%
		Int	9	11	72.7%	39.0%	93.9%
		Int	10	11	36.3%	10.9%	69.2%
		Int	11	12	41.6%	15.1%	72.3%
		Int	14	12	33.3%	9.9%	65.1%
		Int	25	11	45.4%	16.7%	76.6%
Enterococcus	Gentamycin	Min	1	12	0.0%	0%	26.5%
		Min	6	10	0.0%	0%	30.8%
		Min	7	12	8.3%	0.2%	38.4%
		Min	8	7	0.0%	0%	41.0%
		Min	9	12	0.0%	0%	26.5%
		Min	10	12	0.0%	0%	26.5%
		Min	11	11	0.0%	0%	28.5%
		Min	14	12	0.0%	0%	26.5%
		Min	25	12	0.0%	0%	26.5%
		Mod	1	12	16.6%	2.0%	48.4%
		Mod	6	12	0.0%	0%	26.5%
		Mod	7	10	0.0%	0%	30.8%
		Mod	8	8	0.0%	0%	36.9%
		Mod	9	10	10.0%	0.2%	44.5%
		Mod	10	9	0.0%	0%	33.6%
		Mod	11	10	0.0%	0%	30.8%
		Mod	14	9	0.0%	0%	33.6%
		Mod	25	11	0.0%	0%	28.5%
		Int	1	12	8.3%	0.2%	38.4%
		Int	6	11	9.0%	0.2%	41.2%
		Int	7	9	0.0%	0%	33.6%
		Int	8	12	0.0%	0%	26.5%
		Int	9	11	9.0%	0.2%	41.2%
		Int	10	11	9.0%	0.2%	41.2%
		Int	11	12	8.3%	0.2%	38.4%
		Int	14	12	0.0%	0%	26.5%
		Int	25	11	9.0%	0.2%	41.2%
Enterococcus	Kanamycin	Min	1	12	33.3%	9.9%	65.1%

Enterococcus	Kanamycin (continued)	Min	6	10	0.0%	0%	30.8%
		Min	7	12	8.3%	0.2%	38.4%
		Min	8	7	0.0%	0%	41.0%
		Min	9	12	0.0%	0%	26.5%
		Min	10	12	8.3%	0.2%	38.4%
		Min	11	11	0.0%	0%	28.5%
		Min	14	12	8.3%	0.2%	38.4%
		Min	25	12	8.3%	0.2%	38.4%
		Mod	1	12	41.6%	15.1%	72.3%
		Mod	6	12	16.6%	2.0%	48.4%
		Mod	7	10	0.0%	0%	30.8%
		Mod	8	8	12.5%	0.3%	52.6%
		Mod	9	10	20.0%	2.5%	55.6%
		Mod	10	9	11.1%	0.2%	48.2%
		Mod	11	10	50.0%	18.7%	81.2%
		Mod	14	9	0.0%	0%	33.6%
		Mod	25	11	0.0%	0%	28.5%
		Int	1	12	16.6%	2.0%	48.4%
		Int	6	11	9.0%	0.2%	41.2%
		Int	7	9	0.0%	0%	33.6%
		Int	8	12	8.3%	0.2%	38.4%
		Int	9	11	9.0%	0.2%	41.2%
		Int	10	11	9.0%	0.2%	41.2%
		Int	11	12	8.3%	0.2%	38.4%
		Int	14	12	16.6%	2.0%	48.4%
		Int	25	11	27.2%	6.0%	60.9%
Enterococcus	Lincomycin	Min	1	12	100%	73.5%	100%
		Min	6	10	100%	69.1%	100%
		Min	7	12	91.6%	61.5%	99.7%
		Min	8	7	85.7%	42.1%	99.6%
		Min	9	12	91.6%	61.5%	99.7%
		Min	10	12	100%	73.5%	100%
		Min	11	11	90.9%	58.7%	99.7%
		Min	14	12	100%	73.5%	100%
		Min	25	12	100%	73.5%	100%
		Mod	1	12	100%	73.5%	100%
		Mod	6	12	75.0%	42.8%	94.5%
		Mod	7	10	80.0%	44.3%	97.4%
		Mod	8	8	100%	63.0%	100%
		Mod	9	10	100%	69.1%	100%
		Mod	10	9	100%	66.3%	100%
		Mod	11	10	100%	69.1%	100%
		Mod	14	9	100%	66.3%	100%
		Mod	25	11	90.9%	58.7%	99.7%
		Int	1	12	100%	73.5%	100%
		Int	6	11	90.9%	58.7%	99.7%
		Int	7	9	88.8%	51.7%	99.7%

Enterococcus	Lincomycin (continued)	Int	8	12	91.6%	61.5%	99.7%
		Int	9	11	100%	71.5%	100%
		Int	10	11	100%	71.5%	100%
		Int	11	12	100%	73.5%	100%
		Int	14	12	83.3%	51.5%	97.9%
		Int	25	11	90.9%	58.7%	99.7%
Enterococcus	Linezolid	Min	1	12	0%	0%	26.5%
		Min	6	10	0%	0%	30.8%
		Min	7	12	0%	0%	26.5%
		Min	8	7	0%	0%	41.0%
		Min	9	12	0%	0%	26.5%
		Min	10	12	0%	0%	26.5%
		Min	11	11	0%	0%	28.5%
		Min	14	12	0%	0%	26.5%
		Min	25	12	0%	0%	26.5%
		Mod	1	12	0%	0%	26.5%
		Mod	6	12	0%	0%	26.5%
		Mod	7	10	0%	0%	30.8%
		Mod	8	8	0%	0%	36.9%
		Mod	9	10	0%	0%	30.8%
		Mod	10	9	0%	0%	33.6%
		Mod	11	10	0%	0%	30.8%
		Mod	14	9	0%	0%	33.6%
		Mod	25	11	0%	0%	28.5%
		Int	1	12	0%	0%	26.5%
		Int	6	11	0%	0%	28.5%
		Int	7	9	0%	0%	33.6%
		Int	8	12	0%	0%	26.5%
		Int	9	11	0%	0%	28.5%
		Int	10	11	0%	0%	28.5%
		Int	11	12	0%	0%	26.5%
		Int	14	12	0%	0%	26.5%
		Int	25	11	0%	0%	28.5%
Enterococcus	Nitrofurantoin	Min	1	12	0%	0%	26.5%
		Min	6	10	10.0%	0.2%	44.5%
		Min	7	12	0%	0%	26.5%
		Min	8	7	0%	0%	41.0%
		Min	9	12	0%	0%	26.5%
		Min	10	12	0%	0%	26.5%
		Min	11	11	0%	0%	28.5%
		Min	14	12	16.6%	2.0%	48.4%
		Min	25	12	8.3%	0.2%	38.4%
		Mod	1	12	16.6%	2.0%	48.4%
		Mod	6	12	0%	0%	26.5%
		Mod	7	10	20.0%	2.5%	55.6%
		Mod	8	8	0%	0%	36.9%
		Mod	9	10	20.0%	2.5%	55.6%

Enterococcus	Nitrofurantoin (continued)	Mod	10	9	11.1%	0.2%	48.2%
		Mod	11	10	10.0%	0.2%	44.5%
		Mod	14	9	11.1%	0.2%	48.2%
		Mod	25	11	18.1%	2.2%	51.7%
		Int	1	12	0%	0%	26.5%
		Int	6	11	0%	0%	28.5%
		Int	7	9	0%	0%	33.6%
		Int	8	12	0%	0%	26.5%
		Int	9	11	0%	0%	28.5%
		Int	10	11	0%	0%	28.5%
		Int	11	12	0%	0%	26.5%
		Int	14	12	8.3%	0.2%	38.4%
		Int	25	11	27.2%	6.0%	60.9%
Enterococcus	Penicillin	Min	1	12	0%	0%	26.5%
		Min	6	10	0%	0%	30.8%
		Min	7	12	0%	0%	26.5%
		Min	8	7	0%	0%	41.0%
		Min	9	12	8.3%	0.2%	38.4%
		Min	10	12	0%	0%	26.5%
		Min	11	11	0%	0%	28.5%
		Min	14	12	0%	0%	26.5%
		Min	25	12	0%	0%	26.5%
		Mod	1	12	8.3%	0.2%	38.4%
		Mod	6	12	0%	0%	26.5%
		Mod	7	10	0%	0%	30.8%
		Mod	8	8	0%	0%	36.9%
		Mod	9	10	0%	0%	30.8%
		Mod	10	9	0%	0%	33.6%
		Mod	11	10	0%	0%	30.8%
		Mod	14	9	0%	0%	33.6%
		Mod	25	11	0%	0%	28.5%
		Int	1	12	8.3%	0.2%	38.4%
		Int	6	11	0%	0%	28.5%
		Int	7	9	0%	0%	33.6%
		Int	8	12	0%	0%	26.5%
		Int	9	11	0%	0%	28.5%
		Int	10	11	9.0%	0.2%	41.2%
		Int	11	12	0%	0%	26.5%
		Int	14	12	8.3%	0.2%	38.4%
		Int	25	11	18.1%	2.2%	51.7%
Enterococcus	Quin / Dalf	Min	1	12	91.6%	61.5%	99.7%
		Min	6	10	40.0%	12.1%	73.7%
		Min	7	12	16.6%	2.0%	48.4%
		Min	8	7	0%	0%	41.0%
		Min	9	12	50.0%	21.0%	78.9%
		Min	10	12	66.6%	34.8%	90.0%
		Min	11	11	63.6%	30.7%	89.0%

Enterococcus	Quin / Dalf (continued)	Min	14	12	83.3%	51.5%	97.9%
		Min	25	12	58.3%	27.6%	84.8%
		Mod	1	12	75.0%	42.8%	94.5%
		Mod	6	12	16.6%	2.0%	48.4%
		Mod	7	10	20.0%	2.5%	55.6%
		Mod	8	8	37.5%	8.5%	75.5%
		Mod	9	10	50.0%	18.7%	81.2%
		Mod	10	9	33.3%	7.4%	70.0%
		Mod	11	10	80.0%	44.3%	97.4%
		Mod	14	9	77.7%	39.9%	97.1%
		Mod	25	11	63.6%	30.7%	89.0%
		Int	1	12	66.6%	34.8%	90.0%
		Int	6	11	45.4%	16.7%	76.6%
		Int	7	9	44.4%	13.7%	78.8%
		Int	8	12	58.3%	27.6%	84.8%
		Int	9	11	72.7%	39.0%	93.9%
		Int	10	11	36.3%	10.9%	69.2%
		Int	11	12	41.6%	15.1%	72.3%
		Int	14	12	41.6%	15.1%	72.3%
		Int	25	11	45.4%	16.7%	76.6%
Enterococcus	Streptomycin	Min	1	12	33.3%	9.9%	65.1%
		Min	6	10	0%	0%	30.8%
		Min	7	12	8.3%	0.2%	38.4%
		Min	8	7	0%	0%	41.0%
		Min	9	12	0%	0%	26.5%
		Min	10	12	8.3%	0.2%	38.4%
		Min	11	11	9.0%	0.2%	41.2%
		Min	14	12	8.3%	0.2%	38.4%
		Min	25	12	8.3%	0.2%	38.4%
		Mod	1	12	41.6%	15.1%	72.3%
		Mod	6	12	16.6%	2.0%	48.4%
		Mod	7	10	0%	0%	30.8%
		Mod	8	8	12.5%	0.3%	52.6%
		Mod	9	10	20.0%	2.5%	55.6%
		Mod	10	9	11.1%	0.2%	48.2%
		Mod	11	10	50.0%	18.7%	81.2%
		Mod	14	9	0%	0%	33.6%
		Mod	25	11	0%	0%	28.5%
		Int	1	12	16.6%	2.0%	48.4%
		Int	6	11	18.1%	2.2%	51.7%
		Int	7	9	0%	0%	33.6%
		Int	8	12	8.3%	0.2%	38.4%
		Int	9	11	9.0%	0.2%	41.2%
		Int	10	11	9.0%	0.2%	41.2%
		Int	11	12	8.3%	0.2%	38.4%
		Int	14	12	25.0%	5.4%	57.1%
		Int	25	11	27.2%	6.0%	60.9%

Enterococcus	Tetracycline	Min	1	12	100.0%	73.5%	100.0%
		Min	6	10	90.0%	55.5%	99.7%
		Min	7	12	66.6%	34.8%	90.0%
		Min	8	7	28.5%	3.6%	70.9%
		Min	9	12	66.6%	34.8%	90.0%
		Min	10	12	66.6%	34.8%	90.0%
		Min	11	11	63.6%	30.7%	89.0%
		Min	14	12	58.3%	27.6%	84.8%
		Min	25	12	91.6%	61.5%	99.7%
		Mod	1	12	100.0%	73.5%	100.0%
		Mod	6	12	41.6%	15.1%	72.3%
		Mod	7	10	50.0%	18.7%	81.2%
		Mod	8	8	87.5%	47.3%	99.6%
		Mod	9	10	80.0%	44.3%	97.4%
		Mod	10	9	55.5%	21.2%	86.3%
		Mod	11	10	100.0%	69.1%	100.0%
		Mod	14	9	66.6%	29.9%	92.5%
		Mod	25	11	72.7%	39.0%	93.9%
		Int	1	12	100.0%	73.5%	100.0%
		Int	6	11	63.6%	30.7%	89.0%
		Int	7	9	77.7%	39.9%	97.1%
		Int	8	12	91.6%	61.5%	99.7%
		Int	9	11	100.0%	71.5%	100.0%
		Int	10	11	100.0%	71.5%	100.0%
		Int	11	12	91.6%	61.5%	99.7%
		Int	14	12	58.3%	27.6%	84.8%
		Int	25	11	81.8%	48.2%	97.7%
Enterococcus	Tigecycline	Min	1	12	0.0%	0%	26.5%
		Min	6	10	0.0%	0%	30.8%
		Min	7	12	0.0%	0%	26.5%
		Min	8	7	0.0%	0%	41.0%
		Min	9	12	0.0%	0%	26.5%
		Min	10	12	0.0%	0%	26.5%
		Min	11	11	9.0%	0.2%	41.2%
		Min	14	12	0.0%	0%	26.5%
		Min	25	12	0.0%	0%	26.5%
		Mod	1	12	0.0%	0%	26.5%
		Mod	6	12	0.0%	0%	26.5%
		Mod	7	10	0.0%	0%	30.8%
		Mod	8	8	0.0%	0%	36.9%
		Mod	9	10	0.0%	0%	30.8%
		Mod	10	9	0.0%	0%	33.6%
		Mod	11	10	0.0%	0%	30.8%
		Mod	14	9	0.0%	0%	33.6%
		Mod	25	11	0.0%	0%	28.5%
		Int	1	12	0.0%	0%	26.5%
		Int	6	11	0.0%	0%	28.5%

Enterococcus	Tigecycline (continued)	Int	7	9	0.0%	0%	33.6%
		Int	8	12	0.0%	0%	26.5%
		Int	9	11	0.0%	0%	28.5%
		Int	10	11	0.0%	0%	28.5%
		Int	11	12	0.0%	0%	26.5%
		Int	14	12	0.0%	0%	26.5%
		Int	25	11	0.0%	0%	28.5%
Enterococcus	Tylosin	Min	1	12	91.6%	61.5%	99.7%
		Min	6	10	30.0%	6.6%	65.2%
		Min	7	12	16.6%	2.0%	48.4%
		Min	8	7	0.0%	0%	41.0%
		Min	9	12	25.0%	5.4%	57.1%
		Min	10	12	41.6%	15.1%	72.3%
		Min	11	11	45.4%	16.7%	76.6%
		Min	14	12	50.0%	21.0%	78.9%
		Min	25	12	50.0%	21.0%	78.9%
		Mod	1	12	66.6%	34.8%	90.0%
		Mod	6	12	25.0%	5.4%	57.1%
		Mod	7	10	10.0%	0.2%	44.5%
		Mod	8	8	50.0%	15.7%	84.3%
		Mod	9	10	50.0%	18.7%	81.2%
		Mod	10	9	33.3%	7.4%	70.0%
		Mod	11	10	70.0%	34.7%	93.3%
		Mod	14	9	66.6%	29.9%	92.5%
		Mod	25	11	36.3%	10.9%	69.2%
		Int	1	12	66.6%	34.8%	90.0%
		Int	6	11	27.2%	6.0%	60.9%
		Int	7	9	44.4%	13.7%	78.8%
		Int	8	12	58.3%	27.6%	84.8%
		Int	9	11	72.7%	39.0%	93.9%
		Int	10	11	36.3%	10.9%	69.2%
		Int	11	12	50.0%	21.0%	78.9%
		Int	14	12	33.3%	9.9%	65.1%
		Int	25	11	45.4%	16.7%	76.6%
Enterococcus	Vancomycin	Min	1	12	0%	0%	26.5%
		Min	6	10	0%	0%	30.8%
		Min	7	12	0%	0%	26.5%
		Min	8	7	0%	0%	41.0%
		Min	9	12	0%	0%	26.5%
		Min	10	12	0%	0%	26.5%
		Min	11	11	0%	0%	28.5%
		Min	14	12	0%	0%	26.5%
		Min	25	12	0%	0%	26.5%
		Mod	1	12	0%	0%	26.5%
		Mod	6	12	0%	0%	26.5%
		Mod	7	10	0%	0%	30.8%
		Mod	8	8	0%	0%	36.9%

Enterococcus	Vancomycin (continued)	Mod	9	10	0%	0%	30.8%
		Mod	10	9	0%	0%	33.6%
		Mod	11	10	0%	0%	30.8%
		Mod	14	9	0%	0%	33.6%
		Mod	25	11	0%	0%	28.5%
		Int	1	12	0%	0%	26.5%
		Int	6	11	0%	0%	28.5%
		Int	7	9	0%	0%	33.6%
		Int	8	12	0%	0%	26.5%
		Int	9	11	0%	0%	28.5%
		Int	10	11	0%	0%	28.5%
		Int	11	12	0%	0%	26.5%
		Int	14	12	0%	0%	26.5%
		Int	25	11	0%	0%	28.5%

Table S3. Number and proportion (95% confidence intervals) of resistant isolates for each *E. coli*-antimicrobial and *Enterococcus sp.*-antimicrobial combination by treatment group and sample week. N = number of pens per treatment group where respective organism was isolated.

	Chlor<br>Cipro<br>Eryth<br>Kanam<br>Linco<br>Nitro<br>Quin<br>Strep<br>Tetra<br>Tylosin	Cipro	Cipro<br>Eryth<br>Genta<br>Kanam<br>Linco<br>Quin<br>Strep<br>Tetra<br>Tylosin	Cipro<br>Eryth<br>Kanam<br>Linco<br>Nitro<br>Pen<br>Quin<br>Strep<br>Tetra<br>Tylosin	Cipro<br>Eryth<br>Kanam<br>Linco<br>Nitro<br>Quin<br>Strep<br>Tetra<br>Tylosin	Cipro<br>Eryth<br>Kanam<br>Linco<br>Quin<br>Strep<br>Tetra<br>Tylosin	Cipro<br>Linco<br>Strep<br>Tetra	Cipro<br>Linco<br>Tetra	Eryth<br>Genta<br>Kanam<br>Linco<br>Quin<br>Strep<br>Tetra<br>Tylosin	Eryth<br>Kanam<br>Linco<br>Pen<br>Quin<br>Strep<br>Tetra<br>Tylosin	Erythro<br>Kanam<br>Linco<br>Quin<br>Strep<br>Tetra<br>Tylosin	Eryth<br>Kanam<br>Linco<br>Strep<br>Tetra<br>Tylosin	Eryth<br>Linco
**Minimal Group**													
Enterococcus durans													
Enterococcus faecalis									1	1	2		
Enterococcus faecium		4					1	11			1		1
Enterococcus hirae									1		3		
**Moderate Group**													
Enterococcus faecalis									1		2		
Enterococcus faecium	1	2			1								3
Enterococcus hirae									1		4	2	
**Intensive Group**													
Enterococcus durans													
Enterococcus faecalis									4		2		
Enterococcus faecium		1		3			1	1			1		
Enterococcus hirae			1			1		1	1	1	1		
Enterococcus villorum													

(Continued)	Eryth Linco Nitro Quin Tetra Tylosin	Eryth Linco Pen Quin Tetra Tylosin	Eryth Linco Quin	Eryth Linco Quin Tetra Tige Tylosin	Eryth Linco Quin Tetra Tylosin	Linco	Linco Nitro	Linco Nitro Quin	Linco Quin	Linco Quin Tetra	Linco Strep Tetra	Linco Tetra	Linco Tylosin	Nitro	Pan-susceptible
**Minimal Group**															
Enterococcus durans		1			2										
Enterococcus faecalis				1	18										
Enterococcus faecium			2		1	11	1	3	5	4		4			
Enterococcus hirae					8	1						12			
**Moderate Group**															
Enterococcus faecalis					16							1			
Enterococcus faecium	2					9	4	2	1			1		1	3
Enterococcus hirae					11					3		20			
**Intensive Group**															
Enterococcus durans					2							1			
Enterococcus faecalis					11							2			
Enterococcus faecium						4	1		3		1	2			5
Enterococcus hirae		1			19					1		27	1		
Enterococcus villorum					1										

Table S4. Distribution of antimicrobial resistance patterns (number of isolates) by *Enterococcus* species and treatment group. *Antimicrobial key:* Chlor = chloramphenicol, Cipro = ciprofloxacin, Dapto = daptomycin, Eryth = erythromycin, Genta = gentamicin, Kanam = kanamycin, Linco = lincomycin, Linez = linezolid, Nitro = nitrofurantoin, Pen = penicillin, Quin = quinupristin-dalfopristin, Strep = streptomycin, Tetra = tetracycline, Tige = tigecycline, Tylosin = tylosin tartrate, Vanco = vancomycin

Bacterial Species	Antimicrobial	GEE Group + Date + Group*Date + Rep Meas (QIC)	GEE Group + Date + Rep Meas (QIC)	GEE Group + Rep Meas (QIC)	GEE Date + Rep Meas (QIC)
E.coli	Amoxicillin/Clavulanic Acid			78.33	
E.coli	Ampicillin	432.2	414.37	405.8	416.74
E.coli	Azithromycin				
E.coli	Cefoxitin				
E.coli	Ceftiofur				
E.coli	Ceftriaxone				
E.coli	Chloramphenicol		169.59	161.13	166.67
E.coli	Ciprofloxacin				
E.coli	Gentamicin			47.61	
E.coli	Nalidixic Acid				
E.coli	Streptomycin		367.48	364.89	404.07
E.coli	Sulfisoxazole		260.84	251.83	254.21
E.coli	Tetracycline		224.39	211.64	224.77
E.coli	Trimethoprim/Sulfamethoxazole				
Enterococcus	Chloramphenicol				
Enterococcus	Ciprofloxacin			190.66	
Enterococcus	Daptomycin			90.52	
Enterococcus	Erythromycin	403.83	399.9	407.89	396.95
Enterococcus	Gentamycin			90.81	
Enterococcus	Kanamycin		223.27	223.38	220.93
Enterococcus	Lincomycin			135.27	
Enterococcus	Linezolid				
Enterococcus	Nitrofurantoin			139.66	
Enterococcus	Penicillin			67.85	
Enterococcus	Quinupristin/Dalfopristin	394.93	393.6	407.76	391.29
Enterococcus	Streptomycin		236.41	237.44	232.19
Enterococcus	Tetracycline		299.34	317.16	
Enterococcus	Tigecycline				
Enterococcus	Tylosin		394.49	404.84	392.76
Enterococcus	Vancomycin				

Table S5. Quasi Information Criterion (QIC) results for each bacteria-antimicrobial combinations of the four generalized estimating equation models generated.
